# Variability and predictability in human sleep

**DOI:** 10.1093/braincomms/fcaf469

**Published:** 2025-11-29

**Authors:** Marc G Leguia, Christoph Jaehnig, Ellen van Maren, Cecilia Friedrichs-Maeder, Jonas Duun-Henriksen, Troels Wesenberg Kjaer, Athina Tzovara, Kaspar Schindler, Carolina Gutierrez Herrera, Antoine R Adamantidis, Markus H Schmidt, Maxime O Baud

**Affiliations:** Sleep-Wake-Epilepsy Center, Center for Experimental Neurology, NeuroTec, Department of Neurology, Inselspital Bern, University Hospital, University of Bern, 3007 Bern, Switzerland; Department of Engineering, Universitat Pompeu Fabra, 08018 Barcelona, Catalonia, Spain; Sleep-Wake-Epilepsy Center, Center for Experimental Neurology, NeuroTec, Department of Neurology, Inselspital Bern, University Hospital, University of Bern, 3007 Bern, Switzerland; Sleep-Wake-Epilepsy Center, Center for Experimental Neurology, NeuroTec, Department of Neurology, Inselspital Bern, University Hospital, University of Bern, 3007 Bern, Switzerland; Sleep-Wake-Epilepsy Center, Center for Experimental Neurology, NeuroTec, Department of Neurology, Inselspital Bern, University Hospital, University of Bern, 3007 Bern, Switzerland; UNEEG Medical A/S, DK-3450 Allerød, Denmark; UNEEG Medical A/S, DK-3450 Allerød, Denmark; Sleep-Wake-Epilepsy Center, Center for Experimental Neurology, NeuroTec, Department of Neurology, Inselspital Bern, University Hospital, University of Bern, 3007 Bern, Switzerland; Sleep-Wake-Epilepsy Center, Center for Experimental Neurology, NeuroTec, Department of Neurology, Inselspital Bern, University Hospital, University of Bern, 3007 Bern, Switzerland; Sleep-Wake-Epilepsy Center, Center for Experimental Neurology, NeuroTec, Department of Neurology, Inselspital Bern, University Hospital, University of Bern, 3007 Bern, Switzerland; Sleep-Wake-Epilepsy Center, Center for Experimental Neurology, NeuroTec, Department of Neurology, Inselspital Bern, University Hospital, University of Bern, 3007 Bern, Switzerland; Sleep-Wake-Epilepsy Center, Center for Experimental Neurology, NeuroTec, Department of Neurology, Inselspital Bern, University Hospital, University of Bern, 3007 Bern, Switzerland; Sleep-Wake-Epilepsy Center, Center for Experimental Neurology, NeuroTec, Department of Neurology, Inselspital Bern, University Hospital, University of Bern, 3007 Bern, Switzerland

**Keywords:** sleep, sub-scalp EEG, dynamic time warping, NREM, REM

## Abstract

The quality of sleep and its cognitive benefits rely on the cyclic alternance of two distinct sleep stages associated (REM) or not (NREM) with rapid-eye-movements. The ability to predict shifts in sleep stages could help design future interventions in sleep medicine, but it remains unknown how robust the NREM-REM sleep architecture may be for a given individual over many successive nights. We sought to characterize the individual variability and test the predictability of healthy human sleep recorded longitudinally over unprecedented durations (weeks). Based on ultra-long-term sub-scalp electroencephalographic recordings from a newly available, minimally invasive device, we characterized sleep cycles in eight healthy subjects over a median of 30 consecutive days. We first decomposed EEG signals into five frequency bands (δ, θ, α, σ and β) using a multi-taper time-frequency transform. Second, we quantified variability in sleep spectral composition and predictability in sleep stage transitions based on unsupervised and supervised learning methods, respectively. Using dynamic time warping, we quantified the dissimilarity (*D*) between pairs of nights, showing that it was lower within (*D* = 2.5 ± 0.7) than across subjects (*D* = 4.1 ± 0.5, *P* < 0.001). Further, we extracted archetypal sleep patterns, which are most representative of an individual’s NREM-REM spectral architecture. Based on the found interplay between δ and σ power bands modeled in a generalized linear model, we predicted transitions from NREM to REM two to four minutes in advance with high accuracy (area under the receiver operating characteristic curve = 0.88). Taken together, these results show that sleep is variable over consecutive nights in healthy subjects but that core dynamics in sleep oscillations are consistently shared across individuals. As a translational outlook, the predictability of certain sleep transitions affords the means to anticipate pathological symptoms specific of a given sleep stage.

## Teaser

Leguia *et al*. characterize individual sleep cycles repeating over a month in eight participants wearing a sub-scalp EEG recording device. Although the symphony of sleep waves varied across participants, shared dynamics at sleep stage transitions were found. Specifically, the authors show that transitions from non-rapid-eye movement to rapid-eye movement sleep can be predicted minutes in advance, promising future clinical applications.

## Graphical Abstract

**Graphical Abstract:**
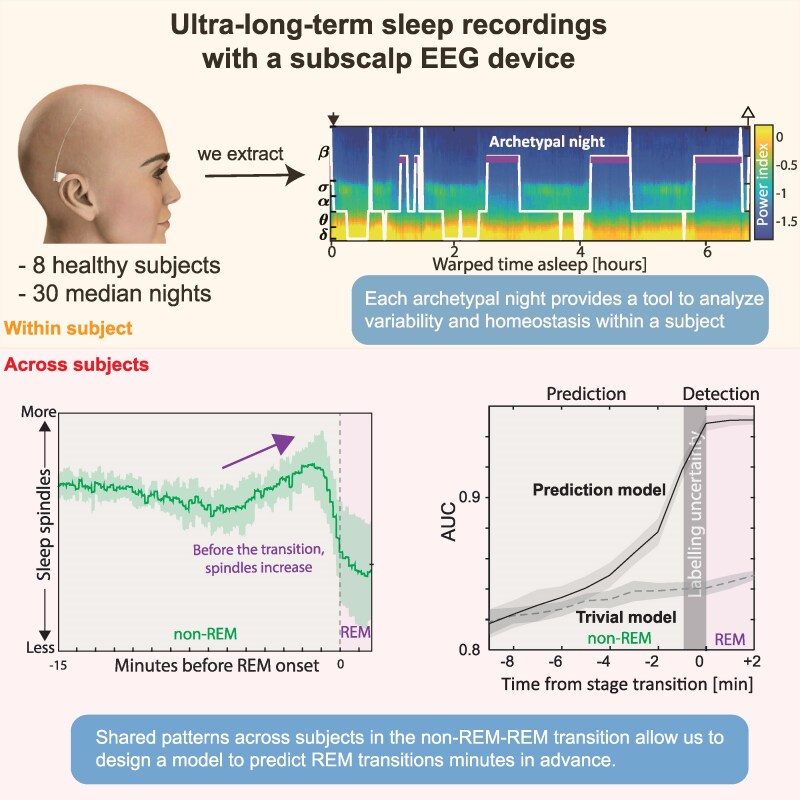

## Introduction

Since the first recording of brain oscillations during sleep,^1^ tracking sleep has evolved into widespread interest in contemporary society. Indeed, a qualitative night of sleep has a refreshing power on an individual’s daytime cognitive and physical well-being.^2^ Currently, measuring sleep EEG in a sleep laboratory is the central method of sleep medicine and research. In the last decade though, new technologies have emerged aiming at measuring sleep at home,^3^ enabling rapid accumulation of sleep data in health and disease. Among them, wearable EEG has the potential to longitudinally track the latent sleep architecture of an individual by recording sleep oscillations night after night from the forehead,^4^ from behind^5^ or within^6^ the ear or from beneath the scalp.^7^

The original classification of sleep stages relies on the fundamental recognition of two distinct types of sleep, associated with rapid (REM) or non-rapid-eye movements (NREM or *N*).^8,9^ Over the decades, the practical needs of sleep medicine have consolidated a rigid system of mutually exclusive categories: wake, N1, N2, N3 and REM, and related hypnograms. Typically, sleep is quantified with ‘static’ statistics in aggregate, for example, the duration and proportion of different sleep stages in a given night.^10^ Such statistics compress observations over time into one number for a whole night of sleep. Yet, any practitioner of sleep scoring recognizes that sleep stages are inter-penetrable and current methods do not account for the ‘dynamic’ nature of the NREM-REM cycle^10,11^ or probabilities of state transition therein.^12,13^ Technological means to capture and predict such sleep dynamics could have clinical applicability, for example by tailoring treatments to prevent NREM parasomnias^14^ or REM sleep behaviour disorder.^15^

Fundamentally, an unbiased, data-driven representation of the spectral continuum in sleep,^12^ learned by a machine, could better account for its variable spectral composition and recurrent temporal dynamics. Reflecting underlying network connectivity,^16^ well-established sleep oscillations include spindles (captured as σ power, highest during N2) and slow waves (captured as δ power, highest during N3). REM sleep, in contrast, is characterized by more variable low-amplitude oscillations, giving rise to a ‘wake-like EEG’. The occurrence of individual oscillations and their synchrony is variable but constrained by the timeframe of the ∼90 min-long NREM-REM cycle. Because access to continuous recordings of sleep over many nights is limited, the variability and predictability of this spectral architecture in healthy sleep is currently unknown.

Our study aimed to uncover the evolution of the spectral composition of sleep over an extended timeframe of one month. To that aim, we recorded sleep in healthy individuals at home, using a novel implantable two-channel sub-scalp EEG (sqEEG) device and learned recurring patterns using interpretable methods. Here, we first derived the archetypal sleep patterns of an individual, using a dynamic algorithm that flexibly accounts for spectral variations during sleep. We then demonstrated the predictability of key transitions between NREM and REM sleep stages, based on recurring spectral patterns shared among individuals.

## Materials and methods

### Subjects

**Figure 1:**
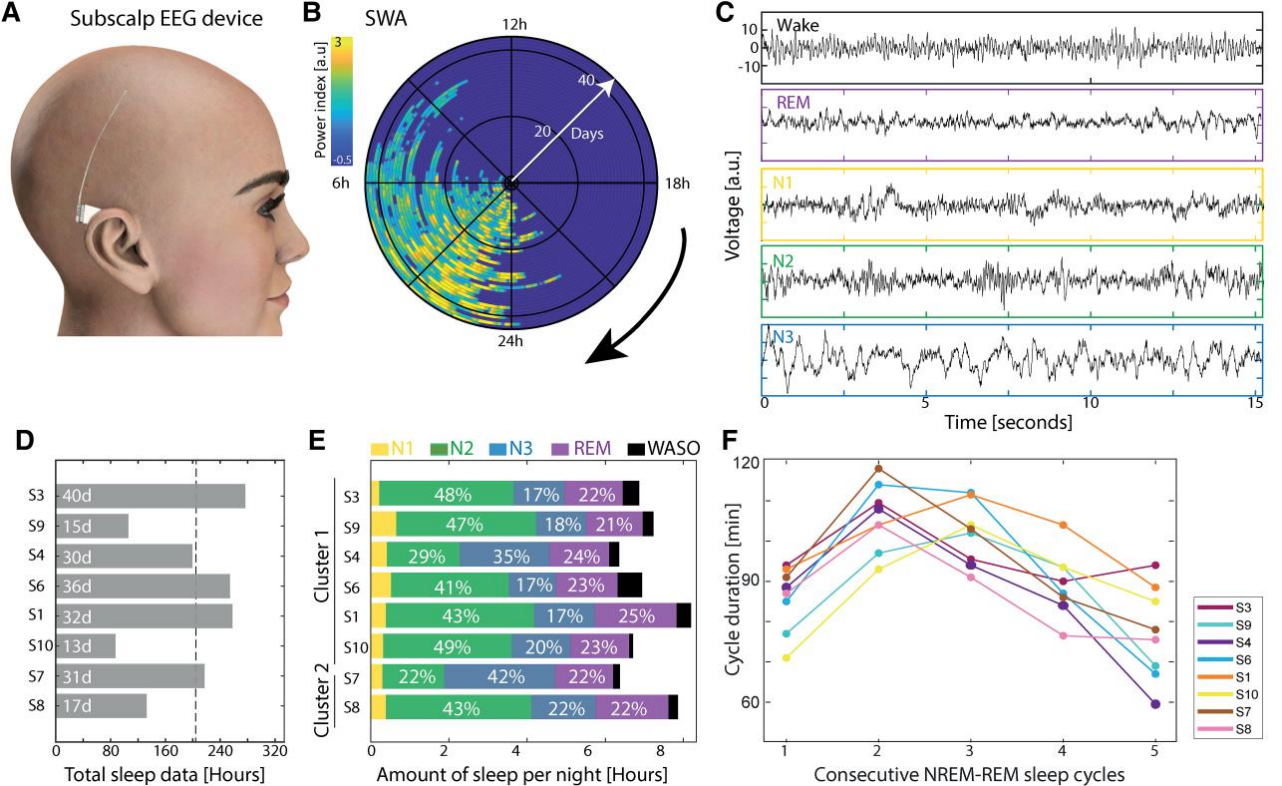
**sqEEG recordings.** (**A**) Schematic of the 24/7 EEG SubQ device (sqEEG) inserted between the scalp and skull to collect sleep and wake EEG signals over weeks. (**B**) Complete dataset of 42 consecutive days and nights recorded in subject 3 shown as Z-scored δ power [1–4 Hz, also referred to as slow wave activity (SWA)] for visualization of sleep periods (wake set to zero). (**C**) Examples of 15s-long sqEEG traces from wake (W), non-REM sleep stage N1, N2 and N3 and REM sleep. (**D**) Total hours of sleep recorded, scored and analysed per subject (inset: the total number of full days recorded). The dashed vertical line is the median across subjects. (**E**) Average duration of night time sleep and percentage of sleep stages per subject. WASO, wake after sleep onset. Clusters from Fig. 2. (**F**) Average duration of successive NREM-REM sleep cycles within subjects.

Healthy volunteers participating in the first trial of a novel sqEEG device (24/7 EEG SubQ, manufacturer: UNEEG medical, Allerød, Denmark, Fig. 1A) were recruited at the Department of Endocrinology, Sydvestjysk Sygehus, Haraldsgade, DK-6700 Esbjerg between 17 January 2015 and 12 January 2017 (NCT02402153). Inclusion criteria into the control group of a diabetes study included healthy adults not using any neuro-active medication, drugs, alcohol or any active medical device. Of 12 recorded subjects, 8 subjects met our inclusion criteria. Four subjects were excluded: three due to discontinuity of data (>50% missing data), and one did not have recognizable sleep oscillations upon extensive visual inspection (see below). All subjects consented to the implantation of the device for data acquisition and *post-hoc* analysis was here performed on anonymized data.

### Data acquisition

sqEEG signals were acquired from one single sub-scalp electrode lead with three contacts with a contact-to-contact distance of 3 cm. The electrode lead was inserted from a retro-auricular incision towards a point 3 cm posterior to the vertex of the head over the parietal bone. Two bipolar derivations were recorded at 207 Hz with the middle electrode contact serving as the reference. The dynamic range is set at 350 µV with a resolution of <1 µV. Built-in filters included a high-pass filter at 0.5 Hz and a 40 dB filter at 48 Hz.

### Visual scoring

All nights in all subjects were visually scored by one of five scorers using an EEG visualization software that displayed a 30 s window along with a 24 h multi-taper spectrogram (all hypnograms in Supplementary Fig. 2). For each subject one night was selected to be scored by all scorers to test the inter-rater agreement, quantified as Cohen’s kappa, obtained for each individual scorer against the plurality of the other scorers. The final Cohen’s Kappa agreement was obtained by averaging individual Cohen’s Kappa. Only nights of sleep longer than 1.5 NREM-REM cycle and without long disconnections were included in the final analysis. Out of the total 295 nights, 81 (27%) were discarded because of night-time disconnections of the device.

### Data pre-processing

sqEEG segments with large artefacts, detected as voltages or line-length higher than ad-hoc thresholds were discarded.

### Time-frequency analysis

Frequency bands of interest were defined as delta (δ, 1–4 Hz), theta (θ, 4–8 Hz), alpha (α, 8–12 Hz), sigma (σ, 12–16 Hz) and beta (β, 16–30 Hz) and derived using a multi-taper spectral power estimate with seven tapers over 12 s without overlap. We also derived periodicity >1 s from the envelope of the σ band (see below).

### Spindle detection

We adapted a published spindle algorithm^20^to our human sqEEG data. From segments scored as N2 or N3, we calculated the envelope (here the absolute power) of the sqEEG signal filtered between 11 and 16 Hz. Candidate spindles were identified as a signal envelope five times higher than the mean value. Their onset and offset were determined when the envelope decreased below three times the mean value. Of the candidate spindles, we discarded those with overlap or a duration >2.5 or <0.5 s.

### Open-ended dynamic time warping

**Figure 2:**
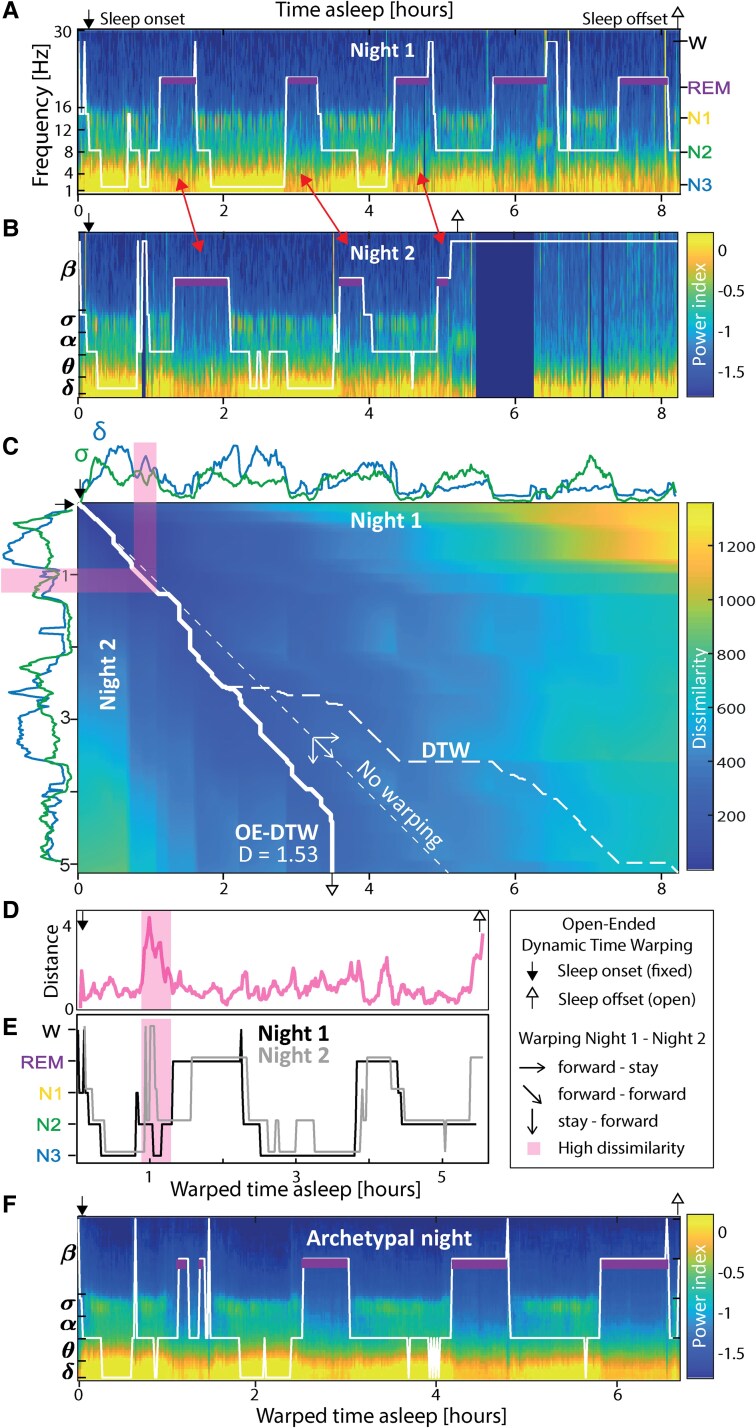
**Dynamical sleep warping and archetypal sleep pattern.** Illustration of the derivation of the archetypal sleep pattern in one example subject 3. (**A**, **B**) Example of within-subject variability in sleep over two consecutive nights with similar spectrograms over the first three cycles, but early awakening at 5 a.m. (missing data related to disconnection, e.g. showering). Hypnograms (white traces) and highlighted REM sleep (purple rectangles) emphasize ‘misalignment’ (red double-arrows) due to different sleep stage duration. (**C**) Distance matrix between the two nights. On top [night 1 from (**A**)] and left [night 2 from (**B**)], normalized δ[1–4Hz] (blue) and σ[12–16 Hz] power (green) are two of the five dimensions along with θ[4–8 Hz], α[8–12 Hz] and β[16–30 Hz] from which the distance (or dissimilarity) matrix is calculated between each pair of time-points from the two nights. Thick and dashed white lines depict the trajectories followed by the OE-DTW and classical DTW algorithms, respectively. OE-DTW allows to match only the first portion of the longer night to account for the shorter second night. Thin dashed line represents the ‘no warping’ line. At each time point (start upper left corner and fixed sleep onset), the path is chosen from one of three possibilities (open arrowheads) such as to minimize the distance. (**D**) The resulting distance along the warping trajectory. The final dissimilarity score is averaged and normalized by the length of the trajectory: here, *D* = 1.53, *D* → 0 for nearly identical nights, and *D* = 4.9 for temporally shuffled nights for that subject. (**E**) Illustration of the final alignment of night 1 and 2 (right and up shifted for visualization puropses). Note that OE-DTW was applied to the spectral bands and not to the hypnograms, leading to an additional source of dissimilarity in the spectral composition, even though the stage may have been scored identically. Highlighted in pink (**D**, **E**), a stage discrepancy in the first cycles of night 1 and 2 with higher focal dissimilarity. (**F**) Corresponding archetypal night extracted using DBA across the 40 nights recorded from subject 3.

Dynamic time warping (DTW) is an algorithm used in a variety of fields^36-39^ to compare and align temporal sequences that may not unfold at the same rate, by minimizing the distance between the two through local realignments, elongations and compressions in time.^40^ Any night of sleep may have a variable number of NREM-REM sleep cycles (e.g. early awakening), typically three to five in our dataset. To account for this, we applied an open-ended (OE) DTW, allowing to match only the first portion of the longer night to the shorter night (Fig. 2). The one fixed point was sleep onset, which was verified visually in all cases. Different nights of sleep with different lengths can thus be warped so that the first few sleep cycles they share align in the warped time-domain. In the process, the amount of warping needed for the best possible alignment is quantified as a dissimilarity index, which is a non-Euclidean distance metric. To do so, we warped each possible, non-adjacent, pair of nights of sleep, using the above-defined five frequency bands (δ, θ, α, σ, β) as input features. Concretely, each night was defined as a 5 × *L_i_* matrix where rows were band-power extracted using a multi-taper transform and *L_i_* was the length of a particular night *i*. The distances between all pairs of timepoints taken from those two nights create a distance matrix *L_i_* × *L_j_*. OE-DTW iteratively chooses the path through the distance matrix to minimize the distance at each branching, and exits when all timepoints of the shorter night have been aligned, thus minimizing the cumulative distance (Fig. 2). Using this procedure, nights with different sleep duration can be compared dynamically, beyond the conventional static statistics.

### DTW barycenter averaging

In addition to pair-wise OE-DTW, we used OE-DTW barycenter averaging (OE-DBA) to extract the barycenter average or ‘archetypal night’ from all nights recorded within a single subject. OE-DBA is a heuristic algorithm that tries to find the archetypal sequence by iteratively minimizing the DTW distance to all of the individual sequences.^41^The key advantage of OE-DBA compared to a mere arithmetic average is to account for the shifts in consecutive nights of sleep with respect to real-world time, which avoids averaging mis-aligned and potentially unrelated sleep stages in the spectral domain. To compute the archetypal night, we define each night as above as a 5 × *L* matrix with *L* being the length of the iterative average. By iterating over all possible nights, the final resulting average is the closest possible to all nights.

### Point process generalized linear models

Point process (PP)-GLMs are powerful, flexible and interpretable statistical model that can evaluate the association between a sequence of events (here, sleep stage transition) and (spectro-)temporal features upon which the event probability may depend. We used a PP-GLM to compute the momentary transition probability between NREM and REM sleep stages (and inversely) based on the five spectral input features defined above. This probability is related to the ‘instantaneous’ rate or conditional intensity function λ(*t*) of the point process, here modelled as:


(1) $$\begin{array}{r} \log\;(\lambda \;|T_{\left\{t-1\right\}}^{\mathrm{stage}},\;P_{\;fb\{t-1\}})\;=\;\beta_{0}+\beta_{T}T_{\left\{t-1\right\}}^{\mathrm{stage}}+\sum \beta_{\;fb}P_{\;fb\{t-1\}} \end{array}$$

Where λ relates to the transition probability with a conditional Poisson distribution, *T*^stage^ is the cumulative time spent in a given stage, and $P_{\;fb\{t-1\}}$ the power in one of the five frequency bands (δ, θ, σ, α and β) here sampled at 1 min duration. To test the null hypothesis that: ‘brain oscillations do not carry any predictive information about upcoming stage transitions’, we compared the above model to the simplified reference model:


$$\;\log\;(\lambda \;|T_{\left\{t-1\right\}}^{\mathrm{stage}})\;=\;\beta_{0}+\beta_{T}^{\;}T_{\left\{t-1\right\}}^{\mathrm{stage}}$$

where only the cumulative time in the stage *T*^stage^ is considered.

In the main analysis, a series of shared model were trained cross-sectionally on half of the nights (107) and tested on the 107 remaining nights across subjects to predict stage transitions at horizons from −8 to +2 min relative to the visually labelled stage tranistion. In a 10-fold cross-validation design, half of the nights were randomly selected for training, the other half for testing. In a supplementary analysis, individual models were trained and tested longitudinally within the same subject. The evaluation of all models is done using the area under the Receiver Operating Characteristic curve (AUC), which evaluates the trade-off between the sensitivity and the specificity in predicting a stage transition at a pre-defined future horizon. In each case, the AUC was statistically compared to the trivial reference model.

### Statistical analysis

Comparisons of dissimilarity measures within or across subjects relied on a Student’s *t*-test. Predictability of sleep stage transitions was assessed with a surrogate-based method. We considered a model to have predictive value—and thus rejected the null hypothesis—only if all distributions of the model’s AUC values exceeded those obtained from trivial or surrogate models.

## Results

We recorded, scored and analysed 214 nights of sleep (13–40 per subject) from eight healthy adults (four women) with median [range] age 35 [25–48] who participated in the UNEEG medical trial of a minimally invasive two-channel sqEEG device (Fig. 1A; Supplementary Table 1). From the unique vantage point of longitudinal sqEEG recordings over many consecutive nights (Fig. 1B), we performed visual analyses of ‘static’ and quantitative analyses of ‘dynamic’ sleep characteristics within and across subjects.

### Conventional sleep characteristics

We visually scored all nights based on the rules of the American Academy of Sleep Medicine adapted for two-channel parietal sqEEG (Fig. 1C; Supplementary Table 2). The median inter-rater agreement was 0.83 (IQR 0.08) among five scorers for one randomly chosen night of sleep per subject (Supplementary Fig. 1). As expected from healthy volunteers, subjects had an average sleep duration between 6 and 8 h, a variable proportion of N2 versus N3, and about 25% REM (Fig. 1D and E; Supplementary Fig. 2).^17^ The mean ± SD length of the NREM-REM cycles was 98 ± 29 min but with a shorter first (92 ± 29 m) and longer second (111 ± 29 m) cycle across subjects (*P* < 0.01, ANOVA, Fig. 1F), in line with other large cohort studies.^18^ These limited analyses based on visual scoring do not capture the richness of spectral patters repeating over the NREM-REM sleep cycle.

### Dynamical sleep warping

Crucially, we next extracted ‘dynamic’ characteristics of sleep from each subject, by learning archetypal sleep patterns and deviations therefrom over the time-frequency continuum. All subsequent analyses were based on a decomposition of the sqEEG signal into five classical frequency bands (δ[1–4 Hz], θ[4–8 Hz], α[8–12 Hz], σ[12–16 Hz] and β[16–30 Hz]), using a multi-taper spectral transform (see methods). This provided a lower-dimensionality feature-based description of spectral changes across the NREM-REM sleep cycle. To dynamically compare the sequence of several NREM-REM sleep cycles between two nights, we used the OE-DTW algorithm that best matched the compared band-pass filtered signals in five dimensions (δ to β, Fig. 2 and methods).

Intuitively, instead of considering time as unfolding at invariable rate, OE-DTW allows to compare two sequences (here two nights of sleep) starting simultaneously but unfolding at variable rates by compressing or elongating time spent at a given stage of the sequence (here a sleep stage, red double arrows in Fig. 2A and B), effectively mapping their alignment coordinates (Fig. 2C). In addition, OE-DTW keeps track of the warping cost, that is the amount of compressions or elongations needed for alignment, effectively quantifying the degree of ‘dissimilarity’ between two nights of sleep (Fig. 2D). As a result, our dissimilarity metric assesses sleep ‘content’ as it reflects differences in spectral composition (e.g. higher δ or α power in a given stage), in detailed sleep architecture (e.g. N3-N2-N3 instead of N3-N2-REM, Fig. 2E), or in stage duration (e.g. longer N2 before transition to N3). In contrast, it is insensitive to the total sleep duration, which can be quantified by simpler means (Fig. 1E).

### Archetypal sleep patterns

To extract the central tendency in sleep patterns, we identified for each subject ‘archetypal’ sleep patterns most representative of the latent sleep architecture (Fig. 2F). Extracting a representative sequence from a set of individual examples is a non-trivial issue. Using OE-DTW barycenter averaging (OE-DBA), we extracted individual archetypal sleep for each subject by computing the ‘barycenter’ with the lowest dissimilarity to all other nights (see methods, Supplementary Figs 3–5). Subject-specific archetypal nights were representative of subjects’ typical 3–4 NREM-REM sleep cycles though the variability around this archetype varied across individuals (Fig. 2F; Supplementary Figs 3 and 4).

### Variability in sleep patterns

**Figure 3:**
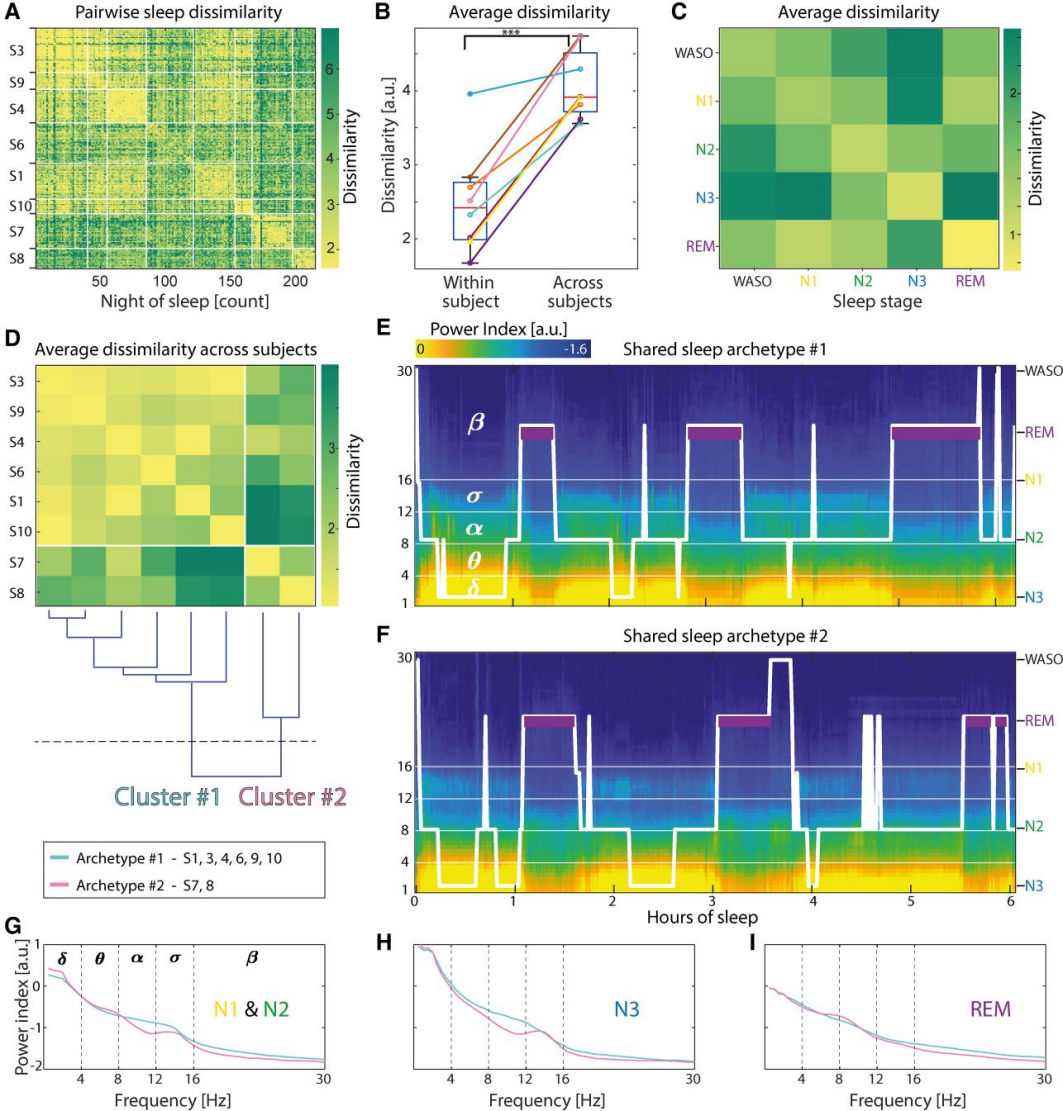
**Variability in sleep patterns.** (**A**) Pair-wise dissimilarity matrix between nights of sleep within and across subjects, calculated using OE-DTW. (**B**) Average pairwise dissimilarity between nights within and across subjects compared with a *t*-test; *** indicates *P* < 0.001. Colour legend per subject in Fig. 1. (**C**) Dissimilarity matrix averaged by epochs visually scored as the same (diagonal cells) or different (off-diagonal) sleep stage(s) across nights and subjects. (**D**) Top: Dissimilarity matrix between individual archetypal nights (shown in Supplementary Fig. 1). Bottom dendrogram: the hierarchical distribution of pairwise average dissimilarity across subjects form twoclusters. (**E, F**) Shared archetypal nights for the six and two subjects in cluster 1 and 2, respectively. (**G-I**) Corresponding power spectra for the two shared archetypal nights from different stages of sleep (G: N1–2, H: N3, I: REM).

Equipped with these methodological advances, we next investigated the variability in sleep patterns within and across subjects, applying OE-DTW to the entire dataset. We found that any pair of nights were relatively similar within individuals (diagonal cells in Fig. 3A; *D* = 2.5 ± 0.7; Supplementary Figs 4 and 5), but more dissimilar (Fig. 3B; *P* = 1.9e^−04^, *t*-test) between two nights taken across individuals (off-diagonal cells in Fig. 3A; *D* = 4.1 ± 0.5). As expected, we found that dissimilarity between two identical or neighbouring sleep stages (e.g. N2-N3) was low, underscoring their spectral similarity, but highest for inconsistency in the aligned sleep stages (e.g. REM versus N3; Fig. 3C). In *post-hoc* analyses, we found that no conventional static features of sleep architecture strongly correlated with our dissimilarity metric (Supplementary Fig. 6). In contrast, day-to-day variations in sleep homeostasis, typically quantified as δ power during sleep, contributed to dissimilarity between nights (Supplementary Figs 7–10). Thus, we developed a method that can capture in one metric the changes in individual spectral dynamics, including the homeostatic regulation of sleep.

### Shared sleep archetypes

We next created a hierarchy and grouped individuals among whom sleep was more resemblant (dendrogram in Fig. 3D). The dissimilarity among subjects within cluster 1 (*D* = 3.4 ± 1.7, subjects 1, 3, 4, 6, 9 and 10) or cluster 2 (*D* = 3.5 ± 1.8, subjects 7 and 8) was lower than between subjects in different clusters (*D* = 4.8 ± 1.8, *P* < 1e-4, *t*-test). Clusters did not show obvious differences in static sleep metrics, such as prevalence of sleep stage or cycle duration (Fig. 1E and F). To qualitatively address how their dynamics differed, we obtained an even more compressed representation of sleep patterns across subjects by deriving ‘shared archetypal sleep patterns’ for individuals in each of the two clusters, that is a representative night with the least dissimilarity to nights from different subjects in the same cluster (Fig. 3E and F). Two noticeable differences in sleep archetype 1 versus 2 were (i) in temporal dynamics: the increasing versus fixed REM duration over subsequent cycles (Fig. 3E and F) and (ii) in spectral composition: the slower spindles in N2-N3 (Fig. 3G-I), a heritable trait.^19^ This illustrates how our unsupervised method can rapidly identify individual recurring ‘archetypal’ patterns and group subjects according to shared sleep dynamics.

### Dynamics in the occurrence of sleep oscillations

**Figure 4:**
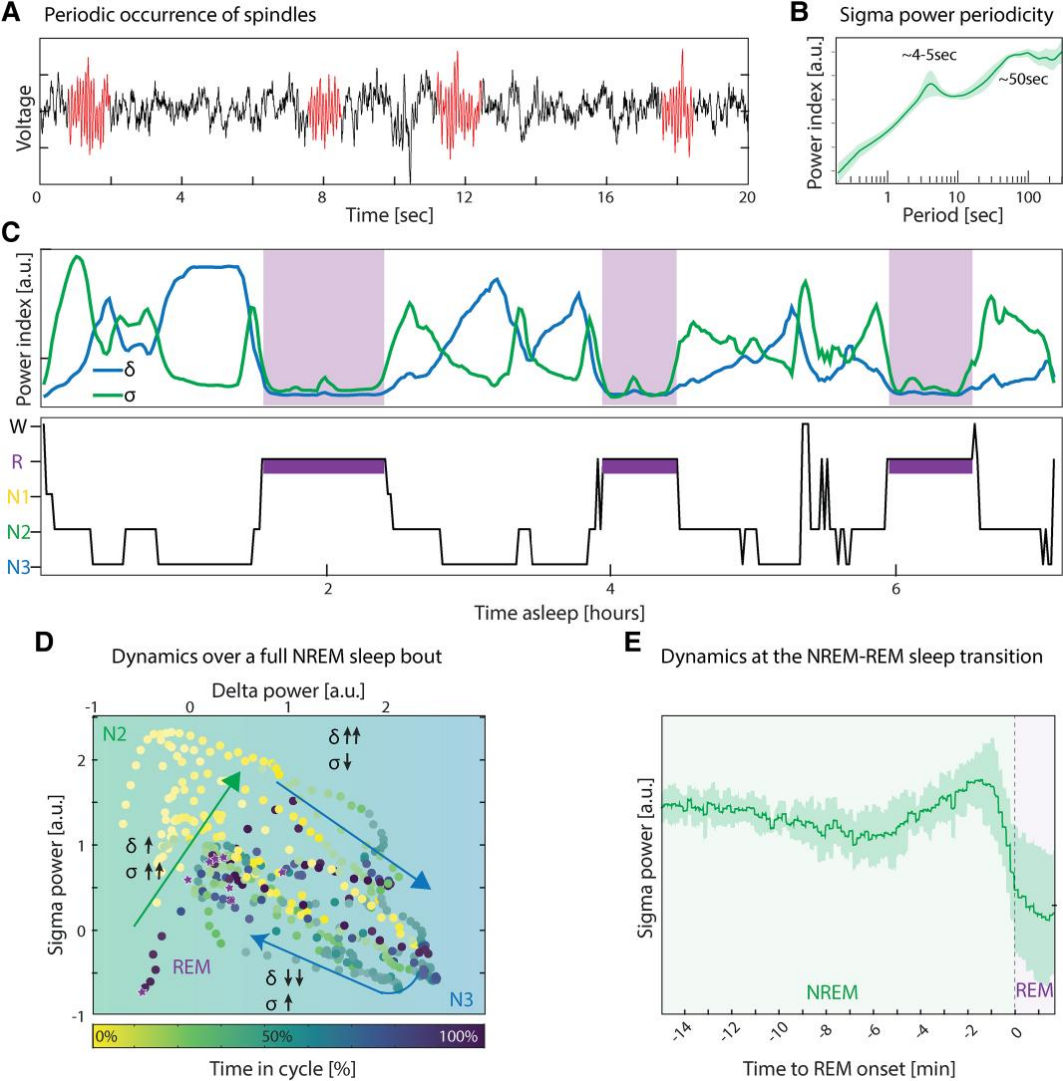
**Dynamics in the occurrence of sleep oscillations.** (**A**) Raw sqEEG trace from NREM stage 2 (N2) sleep containing a sequence of individual spindles (red) at about 4–5 s intervals. (**B**) Power spectrum of the σ power (envelope) revealing slow and infra-slow periodicity (aka as oscillations) of about 4–5 and 40–50 s. (**C**) Archetypal sleep from subject 4 showing varying dynamics and correlations between σ power (capturing sleep spindles) and δ power (capturing sleep slow waves). (**D**) Dynamics in feature space from eight individual archetypal first cycle of NREM-REM sleep. Each datapoint is plotted against average sigma and delta power over 5 min (sliding window of 1 min). Time is normalized to the archetypal duration of the first NREM period and represented as a yellow to blue gradient. Arrows depict the typical trajectory over N2 (yellow–green) and N3 (blue) to REM (violet stars). Transitions to REM sleep mostly occur in the middle of this feature space, but the path to get there may be predictive of an upcoming transition. Yellow (top left) to blue (bottom right) background gradient approximates the feature-space scored as N2 and N3 respectively. (**E**) Average increase in σ power preceding transition from NREM to REM sleep (T_0_) derived from all aligned REM sleep episodes across subjects.

Because our prior cluster analysis had revealed subtle differences in σ peak-power, we next examined the dynamics of slow waves and spindles (Fig. 4A; Supplementary Figs 11–12) within NREM periods and asked how variable they were across subjects. For example, spindles are known to occur with slow (∼4–5 s) and infra-slow periodicity or ‘oscillations’ (∼40–50 s),^19^ which we also observed in our analysis of σ power (Fig. 4B) and of inter-spindle intervals (Supplementary Fig. 13) without noticeable difference between the two clusters (Supplementary Fig. 14). All subjects also showed variable degrees of co-occurrence of spindles with slow waves, with a tendency to occur at the slow wave peak (phase-locking values, median [range] PLV = 0.2 [0.08 to 0.4], Supplementary Fig. 15). Thus, despite the observed variations in σ peak-power (Fig. 3), spindles had similar periodicity and relationship to slow waves across subjects (Fig. 4).

### Dynamics in NREM-REM transitions

Next, we took advantage of archetypal nights to evaluate whether specific σ-δ dynamics may be shared across subjects to signal stage transitions during the NREM-REM cycles (Fig. 4C). During N2, σ power increased sharply up to a maximum, when the trend reversed to a sharp decrease upon transition to N3 (Fig. 4D). During that time, δ power rose steadily until it reached a maximum, at which point a variable amount of σ and δ power was present (Fig. 4D; Supplementary Fig. 16). Thus, during N2, σ and δ are directly correlated, whereas during N3, they are anti-correlated (Supplementary Fig. 15). In the minutes before the transition to REM, we observed a transient sharp rise in σ followed by a strong decrease (Fig. 4E).^20^ This effect was shared across clusters of subjects (Supplementary Figs 14–16). Thus, the dynamics of sleep oscillations during N2-N3 form a continuum,^21^ whereas the transition to REM sleep is sharp, and potentially predictable.

### Predictability in sleep stage transitions

**Figure 5:**
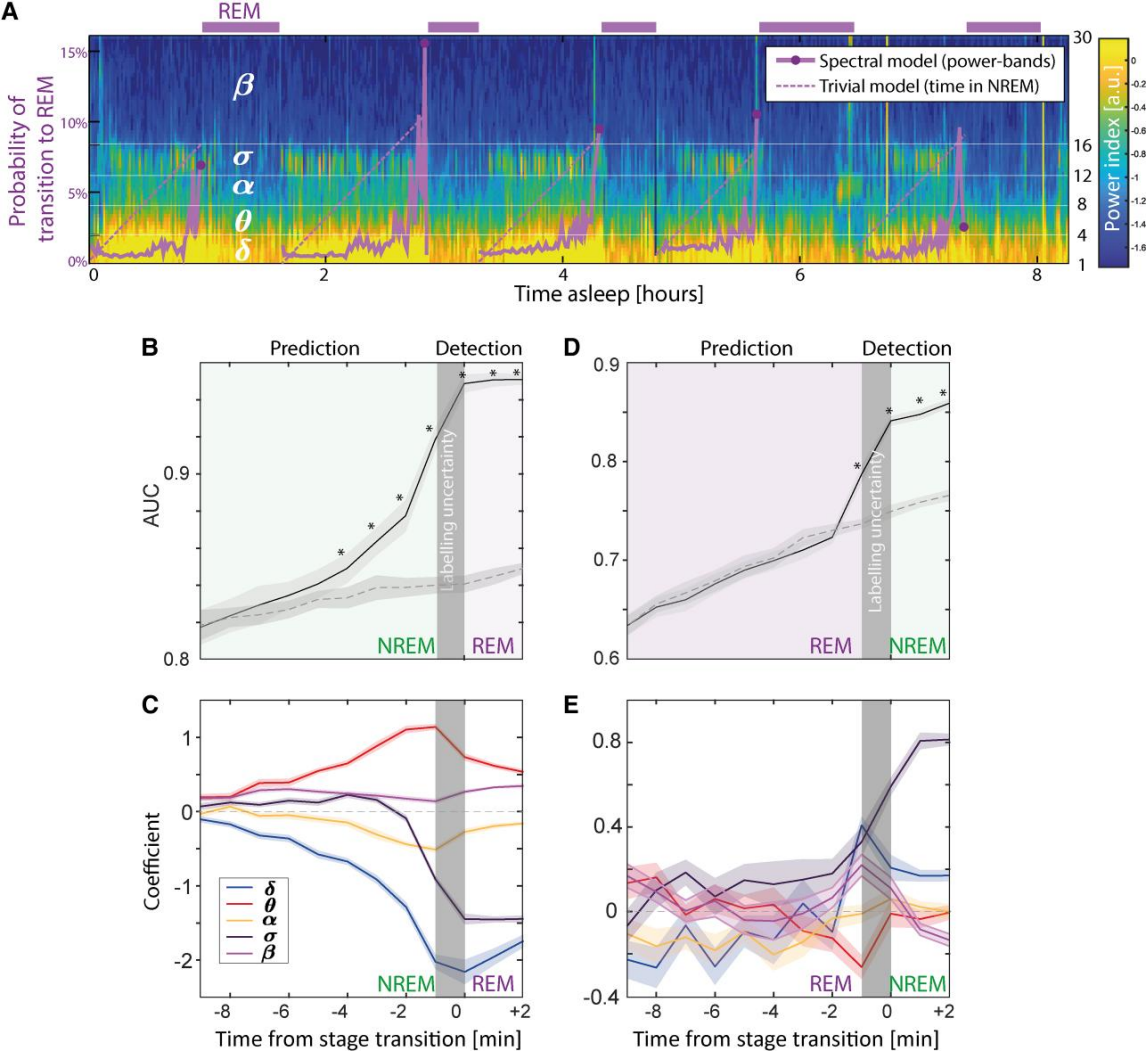
**Predictability in stage transitions.** (**A**) Forecasted probabilities (purple lines) for the transition to REM sleep (purple rectangles) for a single night overlaid on the corresponding spectrogram. The dotted purple line corresponds to a trivial reference model, in which the stage transition probability grows linearly with time spent in NREM sleep. (**B**) Performance of a shared model (across subjects) for the prediction of REM sleep onset over a horizon of minutes quantified as the area-under-the sensitivity-specificity curve (AUC, average ± SD). The grey rectangle highlights our account for human scoring uncertainty (−1 min to T_0_, two scoring windows), as any performance after the true REM onset must be regarded as detection and not prediction. Note that the performance of the spectral model (full line) is superior to that of the trivial model (dotted line) about 4 min before transition. (**C**) Coefficients attributed by the model for the five frequency bands (input features). (**D**, **E**) same as (**B**) and (**C**), but for transitions out of REM into NREM sleep. Note that the performance of the spectral model is not superior to that of the trivial model, also reflected in variable coefficients close to zero (**E**). *Significance in plots b and d is established if the model distribution of AUCs for that time horizon is above the trivial model distribution of AUCs.

To thoroughly evaluate predictability in our uniquely large longitudinal dataset, we trained individual and shared point-process generalized linear models (PP-GLM, methods) to capture the transition probability between sleep stages over future horizons (Fig. 5A). Specifically, we asked whether sqEEG spectral composition may have predictive value above a trivial model (time in preceding stage) and more than 1 min before a visually labelled transition, a preset criterion to account for labelling uncertainty. In a cross-validation design, we trained a shared model over a total of 107 nights across the eight subjects, taking the five power values (δ, θ, α, σ and β) estimated over a 1-min sliding window as input features. The trained model outputs the probability of a given stage transition for a given horizon (from −8 min ahead to 2 min after the transition), which was tested against the ground-truth visually scored transition on unseen test data taken across subjects (total of 107 nights, Fig. 5B-E).

Our PP-GLMs were able to causally predict the transition into REM sleep up to 4 min in advance compared to the trivial reference model (Fig. 5B; supplementary Fig. 17) but not into NREM sleep. Inspection of the model coefficients learned to predict REM sleep showed negative and positive weighting of δ and θ respectively, suggesting the acceleration of sleep oscillations towards REM. In addition, σ weighting flipped from positive to negative two minutes before the onset of REM, confirming observations in mice.^20^ This suggests that the evanescent re-apparition of spindles at the end of NREM (Fig. 4E) may have a specific predictive value for the transition into REM sleep. In a supplementary analysis, we did not observe any predictive value of slow and infraslow variations in spindles for anticipating the transition to REM sleep (Supplementary Fig. 17). Finally, we sought to link our two main findings on sleep variability and predictability. In a *post-hoc* analysis, we found higher predictability for NREM-REM sleep transitions for nights more resemblant to the individual archetypal nights, and lower, but non-trivial predictability for NREM-REM transitions in less typical sleep (Supplementary Fig. 18).

## Discussion

In this work, we quantified the variability and modelled the predictability of sleep dynamics across a cohort of healthy individuals who recorded their sleep night after night with a sqEEG device. The extended duration of this study over an entire month offers a unique perspective, allowing for a nuanced characterization of the temporal dynamics and spectral composition of sleep in real-life conditions. First, our unsupervised method extracted archetypal sleep patterns, beyond the classical static quantifications (e.g. average sleep stage duration), allowing for the comparison of sleep dynamics within and among individuals. Second, we trained a supervised model shared across individuals that can forecast the transition from NREM to REM sleep minutes in advance. These conceptual and methodological advances pave the way to new applications of ultra-long-term EEG with potentially far-reaching implications for tomorrow’s practice in sleep research and medicine.

Historically and to this day, sleep staging requires expert visual labelling of the EEG. Among recently reviewed^23^ machine-learning methods that propose to reduce human labour, our novel method offers certain advantages. Unlike successes in developing automated sleep scoring systems,^23-28^ our ‘unsupervised’ OE-DTW algorithm extracted archetypal sleep patterns from data without relying on staging rules (Figs 2 and 3). For this reason, we believe that OE-DTW may reduce bias compared to Markov chain models of sleep stage transitions^13,29-32^ that are heavily influenced by the scoring of short bouts in a given stage (e.g. a long bout splits in two short bouts if another stage is intercalated). Thus, our approach is unsupervised (no need for training), unparametrized (e.g. stage transitions) and merely highlights resemblance or dissimilarities in spectral composition over time, from which the latent sleep architecture emerges naturally.

For example, we found spectral trajectories over a continuum across N2 and N3 when displaying learned patterns in a two-dimensional feature space (Fig. 4). Further, subtle but consistent increases in σ power (spindles) preceded the transition to REM sleep (Fig. 4), which helped forecast NREM-REM sleep transitions up to 4 min in advance (Fig. 5). Thus, individual archetypal sleep patterns, offer a personalized lens through which to detect anomalies and predict recurring patterns. Across individuals the approach could inform diagnostic criteria indicative of a given sleep disorder. For example, sleep architecture is typically disrupted in patients with narcolepsy^28^ (fragmentation, sleep-onset REM sleep, etc.) but likely to a variable extent on different nights. Within patient, monitoring inter-night variability could help tailor treatment plans, moving beyond the one-size-fits-all approach that characterizes many current interventions.

Finally, our proof-of-concept that REM sleep onset can be forecasted in humans confirms findings in rodents^20,34,35^ and introduces another exciting dimension to sleep research and preventative approaches in sleep medicine (Fig. 5). The integration of real-time monitoring and adaptive learning algorithms may allow to target dysfunctions linked to specific sleep stages, such as REM sleep behaviour disorder.^15^

This study was limited to only eight healthy volunteers. Others propose to deploy non-invasive approaches more broadly, such as wearing a smart-watch during sleep.^22^ While (minimally) invasive, our measurement of longitudinal EEG in the community can surpass peripheral measurements, as it can resolve sleep dynamics characterized by evolving spectral composition over time. Despite limited head coverage and the lack of electrooculography, our approach was well able to recognize NREM and REM sleep, because the OE-DTW algorithm accounts for the overall sleep architecture. Many variables of daily life (e.g. lifestyle and coffee intake) were not available as covariate, but the future strength of digital sqEEG biomarkers lies in their ability to capture recurring patterns beyond minor perturbations.

As we continue to unlock human sleep-waves to monitor health and disease, the integration of real-time EEG monitoring and machine-learning algorithms holds the potential to revolutionize our approach to sleep medicine, neurological and psychiatric disorders. Ultra-long-term monitoring of sleep opens the doors to a future where the variability and predictability of sleep are harnessed for the betterment of human health.

## Supplementary Material

fcaf469_Supplementary_Data

## Data Availability

The raw data belongs to UNEEG medical A/S and cannot be shared by the authors. The sleep scores are available from the corresponding author upon reasonable request.^33^ The code is available on GitHub https://github.com/MGrauLeguia/human_sleep_predictability.
