# Supplementary material for: Variability and predictability in human sleep
Source: Brain Commun. 2025 Nov 29;7(6):fcaf469. doi: 10.1093/braincomms/fcaf469 (PMC12716275; doi:10.1093/braincomms/fcaf469)
Supplement: fcaf469_Supplementary_Data [file fcaf469_supplementary_data.docx]

**Supplementary materials**

**Variability and predictability in human sleep**

In this section, we provide a number of methodological steps that were necessary to attain the main results presented in the main text. We also provide supportive evidence and supplementary analyses.

## Data selection

From a total of 12 subjects, we were able to keep, score and analyze 8 subjects. Reason for exclusion included:

- A low number of recorded nights of sleep < 12 (subject 2)
- Poor quality EEG recordings, rendering the sleep data impossible to score (subject 5)
- A high number of nightly disconnections of the device resulting in > 50% of missing data (subjects 11-12, Supplementary Fig. 1).

For the included subjects, individual nights with nightly disconnections of the device resulting in large portions of missing data had to be discarded. The information of the selected subjects is in Supplementary Table 1.

| **Subject Id** | **Age** | **Sex** |
| --- | --- | --- |
| **1** | 27 | Male |
| **3** | 39 | Female |
| **4** | 32 | Male |
| **6** | 41 | Female |
| **7** | 48 | Female |
| **8** | 25 | Male |
| **9** | 33 | Male |
| **10** | 37 | Female |

Supplementary Table 1. Age and sex for the subjects included in this study.

## Visual scoring

Serving as a reference for all analyses included in the main text, we visually scored all included nights based on a modification of the ASSM criteria. Subscalp EEG recordings were obtained only with three subscalp electrode contacts yielding two bipolar channels, wherein the middle contact was the reference. The location of the electrode contact was approximately on T5/T6 and P3/P4. There were no electrodes to specifically record electrooculography or electromyography, but electromyographic artifacts could be seen in the EEG. For identifying N1 and REM, since we did not have a dedicated EMG channel available, we evaluated the EMG contamination of our available EEG channels, notably by adding a trace with a high-passed filtered signal >50Hz for visual inspection. Scoring rules and example traces are listed in supplementary Table 2.

| **Wake** | | |
| --- | --- | --- |
| **W (quiet)** | >50% alpha rhythm | 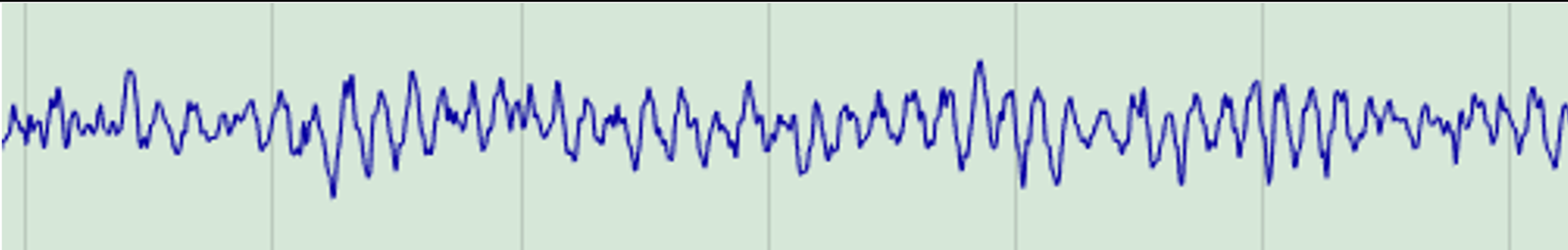  Alpha (throughout, 8-12Hz) with modulation |
| **AW**  **(Active wake)** | Eyes open, low alpha, variable muscle artifacts, movement artifacts | 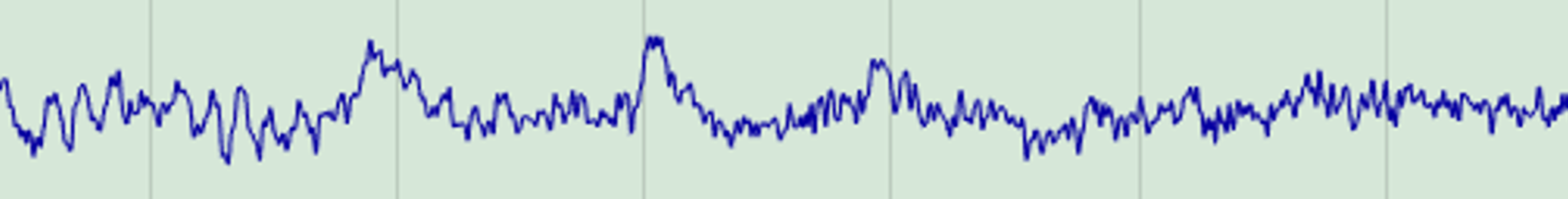Some alpha (left) and Overlaid muscle (right, beta) |
| **E** | Eating artifact (mastication, temporal muscle contraction) | 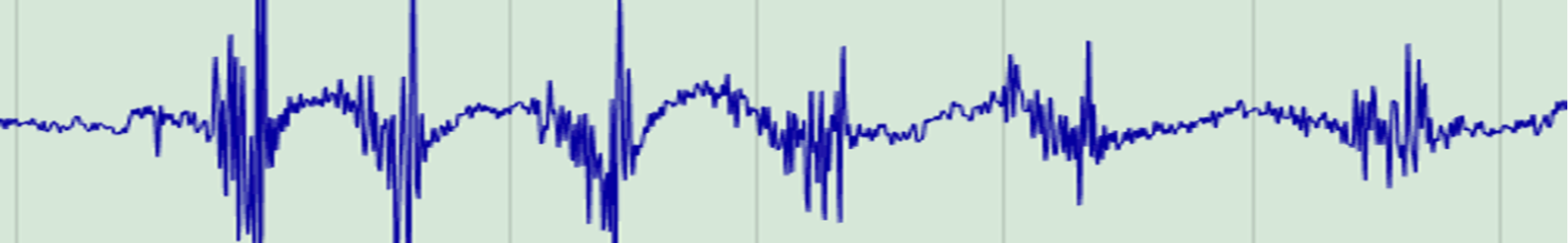 |
| **A** | Technical artifact including disconnections or movements. Typically large deflections, or flat lines. Any 30s epoch with >50% artifact was excluded. Shorter-lasting artifacts were excluded. (here disconnection artifact in the middle). | 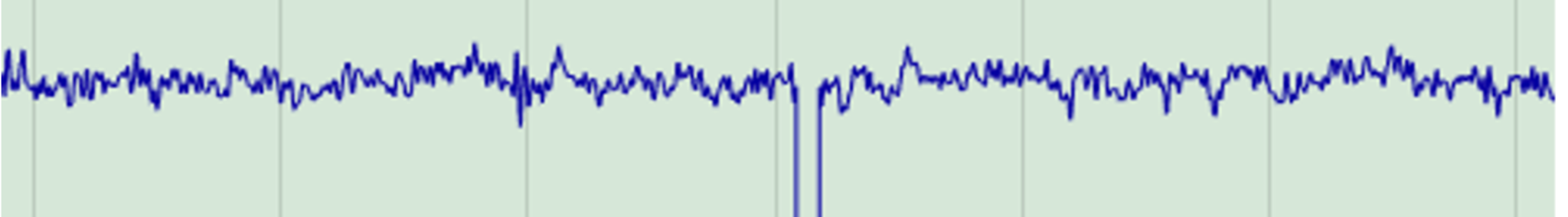Active wake with muscle activity and disconnection artifact (middle) |
| **Sleep** | | |
| **N1** | Onset: >50% alpha drop-out, low-amplitude theta, then Vertex wave (VW, single or group)  Offset to: **N2** or W  Note: VW can be absent. arousal-associated K-complex count for N1. | 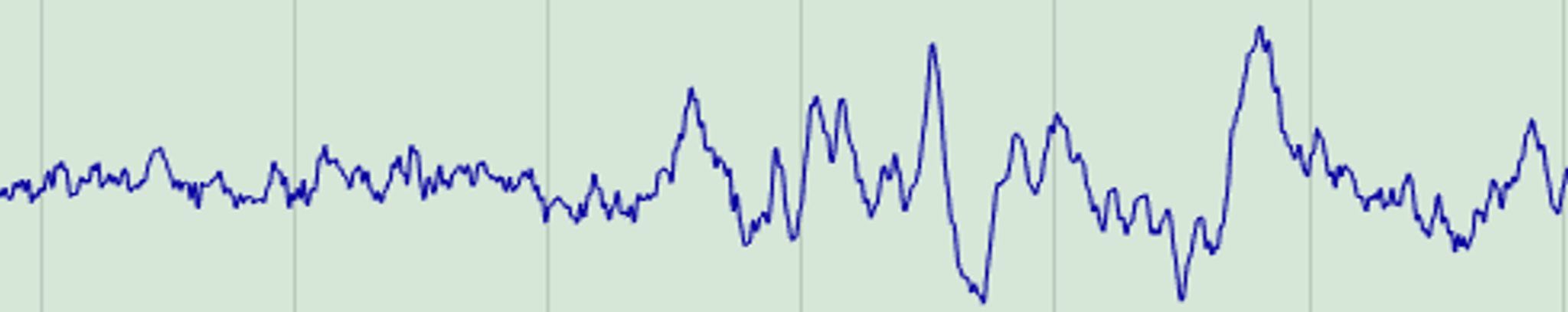Theta (left, 4-7Hz) and Vertex wave in group (right, <500ms) |
| **N2** | Onset: Low EMG, K-complex or spindle in first half of epoch. Scored as long as no transition to other stages. VW can still occur. Theta background, Spindles and K-complexes must not be present on every epoch, but at least every 3 minutes (6 x 30s epochs), otherwise score N1. EMG: Low  Offset to: **N3**, N1, R | 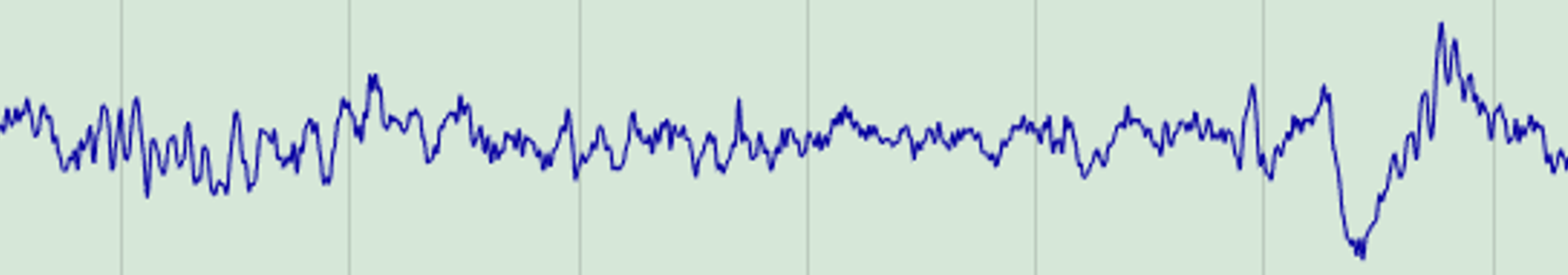Spindle (left, 11-16Hz), Theta (middle) and K-complex (right,≥500ms) |
| **N3** | Onset: > 20% Slow waves (SW) but spindles are still present.  EMG: Low  Offset to: **R**, **N2**, W | 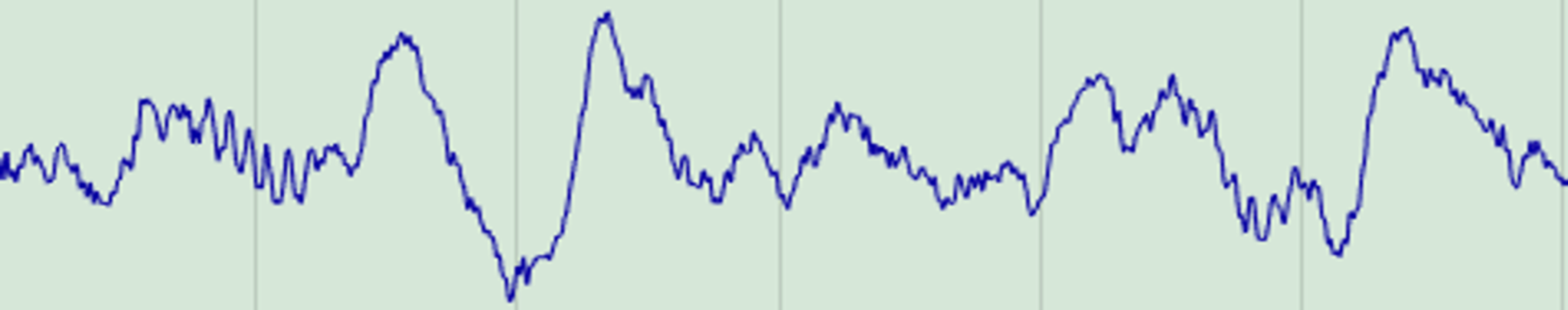Spindle (left) and Slow waves (throughout, 0.5-2Hz, >75 μV) |
| **REM** | Onset: Low-amplitude mixed-frequency with sharp theta and optionally saw-tooth waves. REM alpha is possible, typically 1-2Hz slower than wake alpha.  EMG: Lowest w/ bursts (<250ms)  Offset to: **W, N1, N2** or N3 | 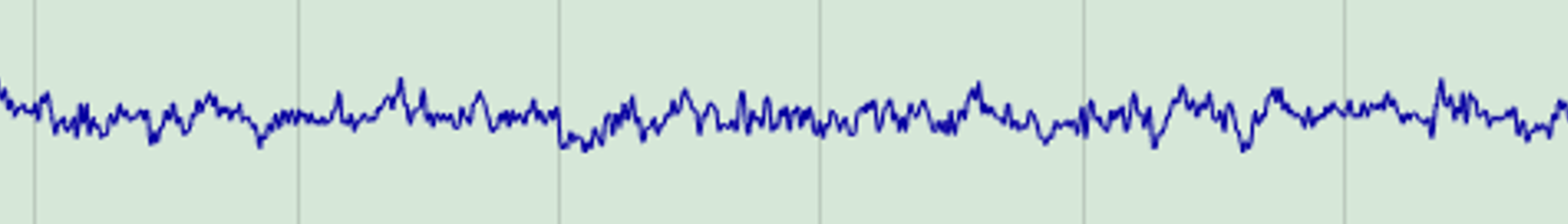Low-amplitude mixed frequencies |
|  |  | 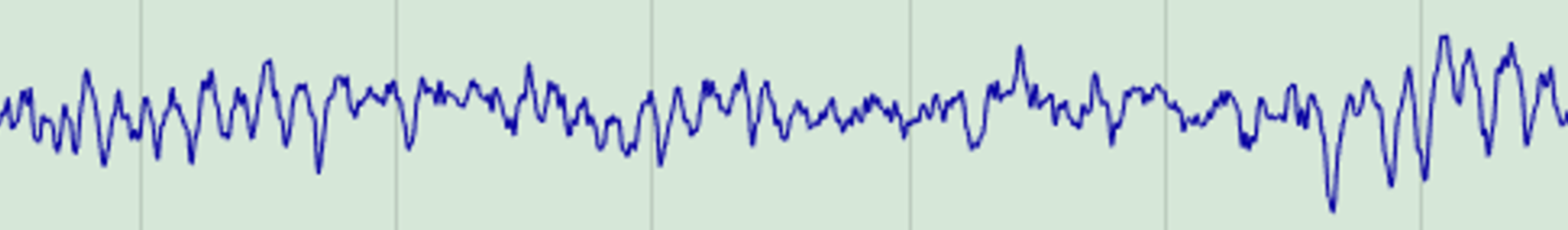Sharp theta (left, 7Hz), mixed (middle), and saw tooth (right, triangular, often serrated, 2-6 Hz) |
|  |  | 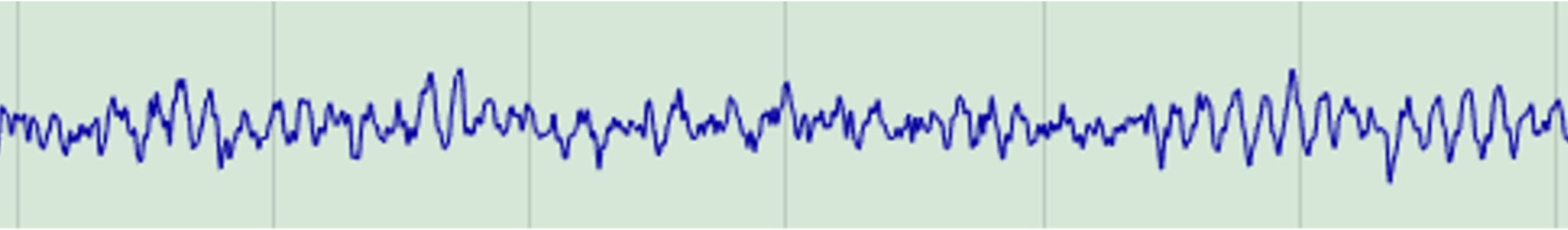Sharp theta (left, 7Hz) and slow alpha (right, 8Hz) |
| **Arousal** | Abrupt frequency change to theta-alpha for > 3sec | **Not scored.** |

**Supplementary Table 2: sqEEG sleep scoring rules.** Chronic sqEEG recordings are scored in 30 seconds epochs, based on AASM rules, adjusted for two-channel parietal EEG. A new stage is given upon occurrence of characteristic elements in the first half of the window. Left: definition of sleep stage. Right: definition of sleep waves.

## Visual scoring consistency

We first verified the consistency in scoring sleep from subscalp EEG by calculating the interscorer agreement, measured as the Cohen’s and Fleiss’s Kappa (Supplementary Fig. 1). Each of five scorers scored the same 12 nights of sleep across subjects. Every two scorers were then compared using the Cohen’s Kappa, defined as


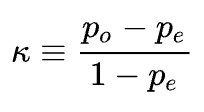


where *Po*, the observed agreement, is put in relation to *Pe*, the agreement obtainable by chance. A Cohen’s Kappa of 1 means a perfect agreement between two scorers, and 0 means no agreement above chance. A Cohen’s Kappa >0.8 is considered near-perfect. To be able to calculate individual and average Cohen’s Kappa, we compared each individual scorer against a consensus of the other scorers. values. The median of Cohen’s Kappa values for *inter*-rater agreement was 0.829, with a mean of 0.813 and an interquartile range of 0.079 (Supplementary Fig. 1a). Agreement among scorers was highest for sleep stages N2, N3, and REM and lowest for N1 (Supplementary Fig. 1b). One night of sleep in two subjects was scored twice by four scorers. The median of Cohen’s Kappa values for *intra*-rater agreement was in the range 0.85 – 0.93, with a mean of 0.88 (not shown).

| **A**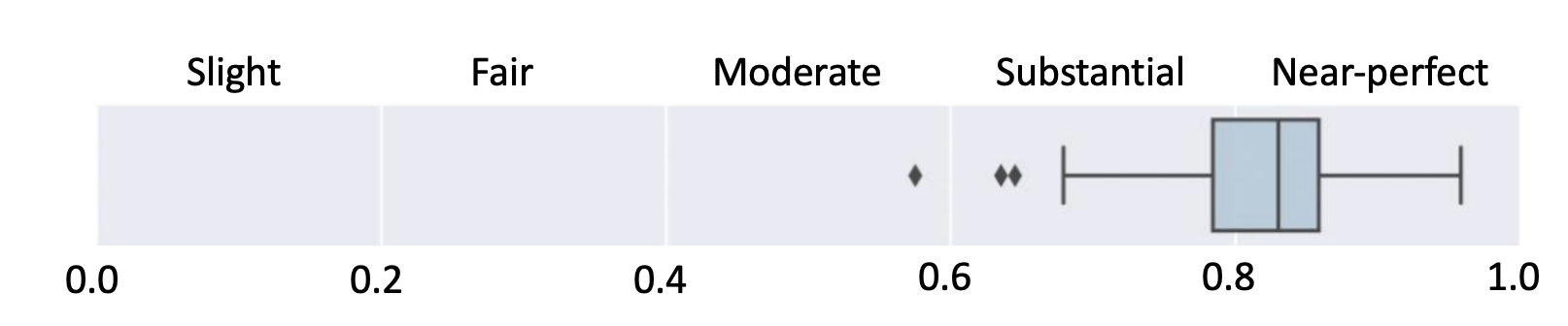 | **Supplementary Fig. 1: Inter-rater agreement. A.** Cohen’s Kappa score for five scorers over 12 nights. **B.** Confusion matrix showing agreement between scorers and  consensus normalized on the scorer-axis. Scorers were compared to the consensus, so that each stage scored in an epoch is compared to the consensus score. |
| --- | --- |
| B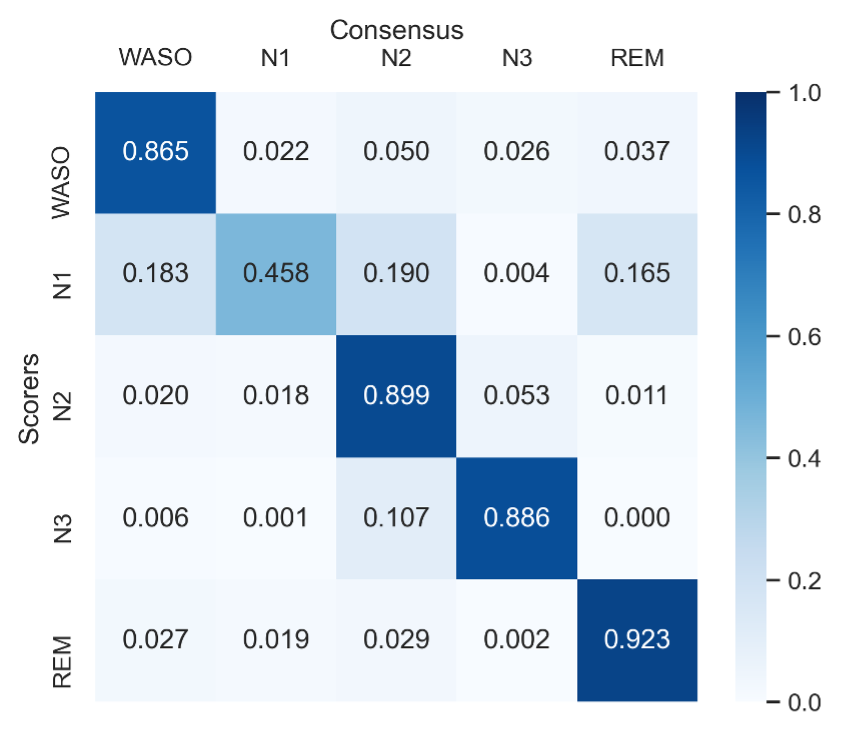 |  |

## Visual scores and statistics

After verifying the inter-rater agreement, each of the five scorers then scored a subset of the nights of sleep. Over the next 14 pages, supplementary Fig. 2 shows all of the data used in this study across 8 subjects. Subjects 2, 5, 11, and 12 were discarded on the basis of insufficient, discontinuous, or poor-quality data. Among the remaining subjects, some scored data show incomplete or discontinuous nights of sleep, which were discarded or used only in some analyses.

**
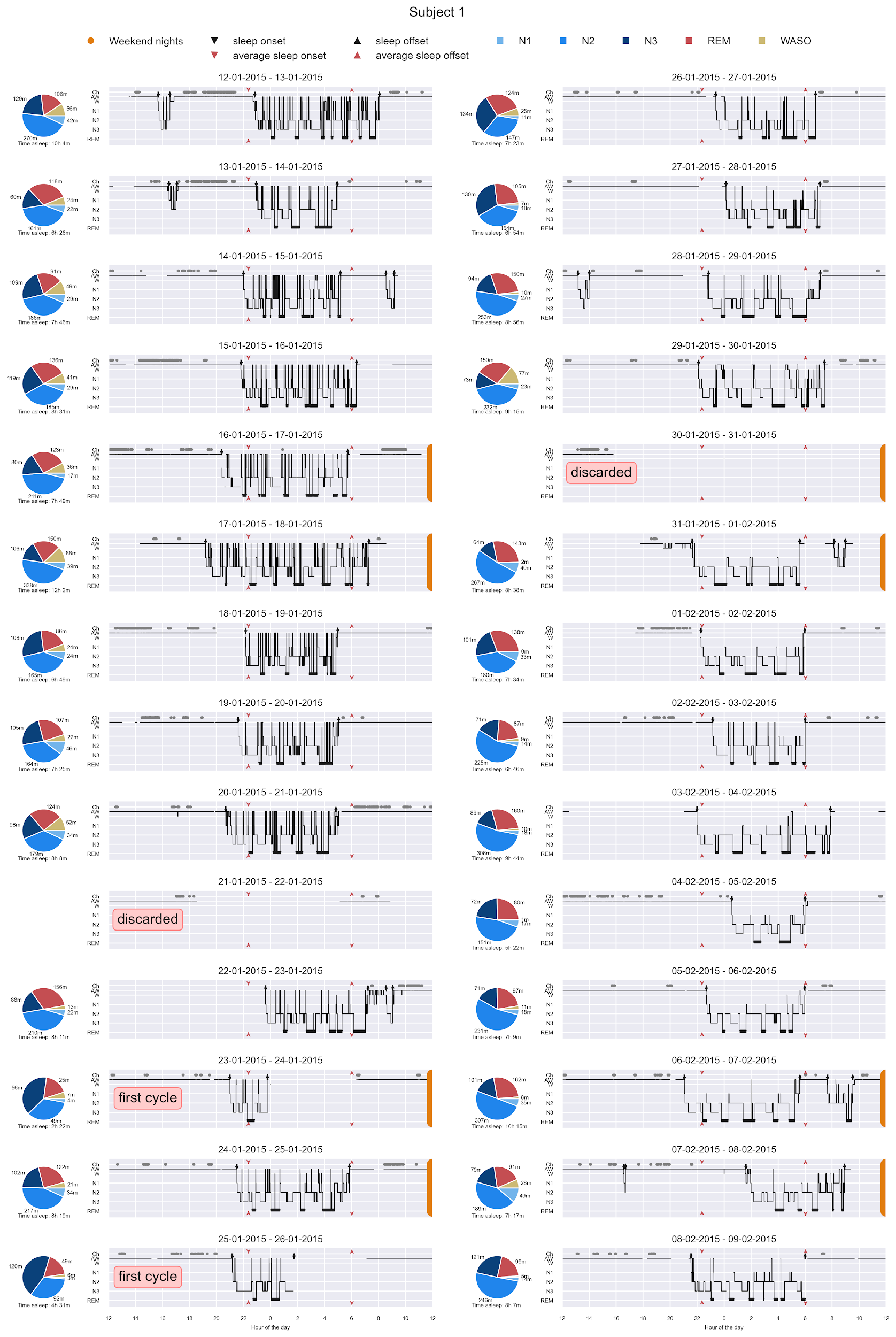
**

**
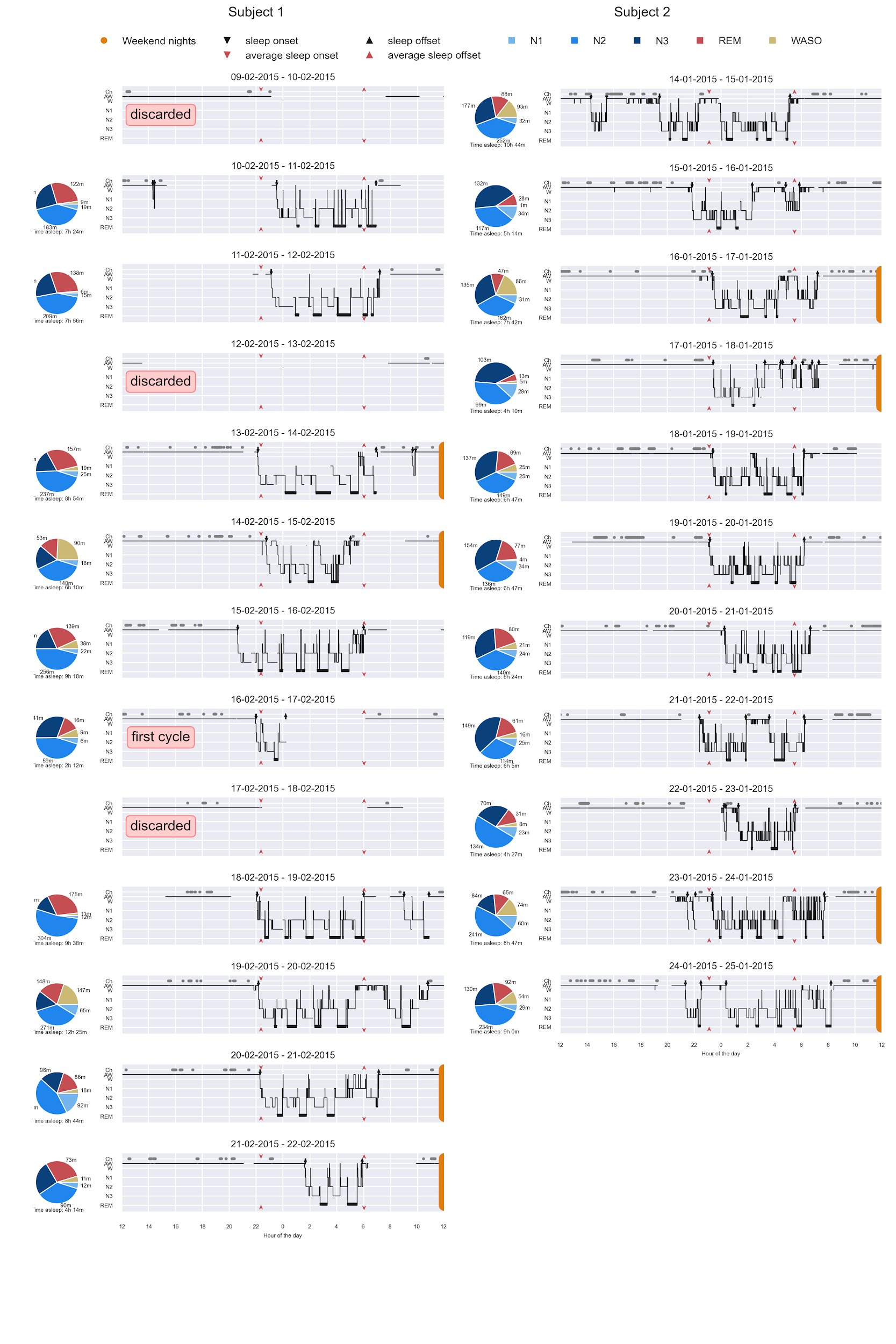
**

**
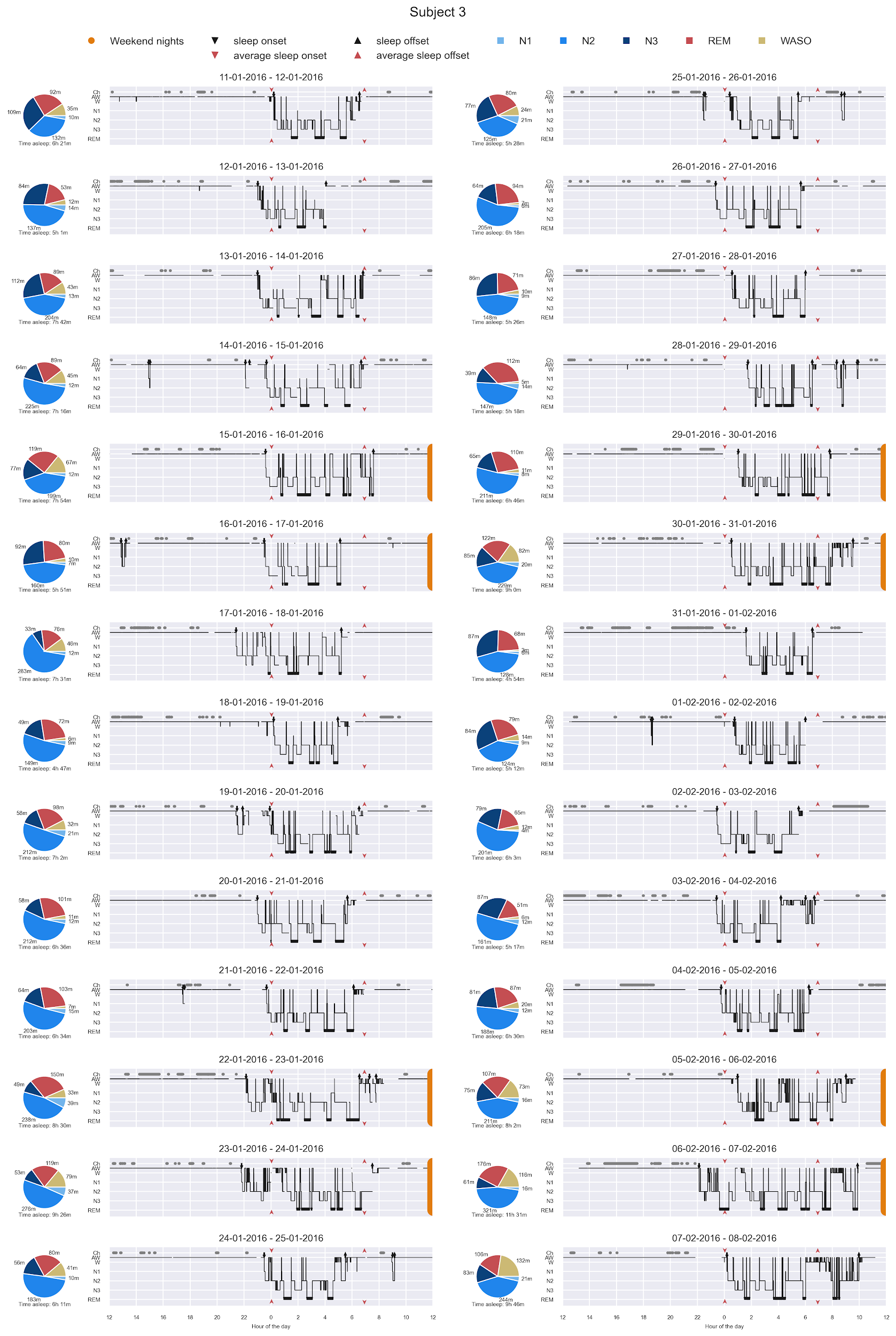
**

**
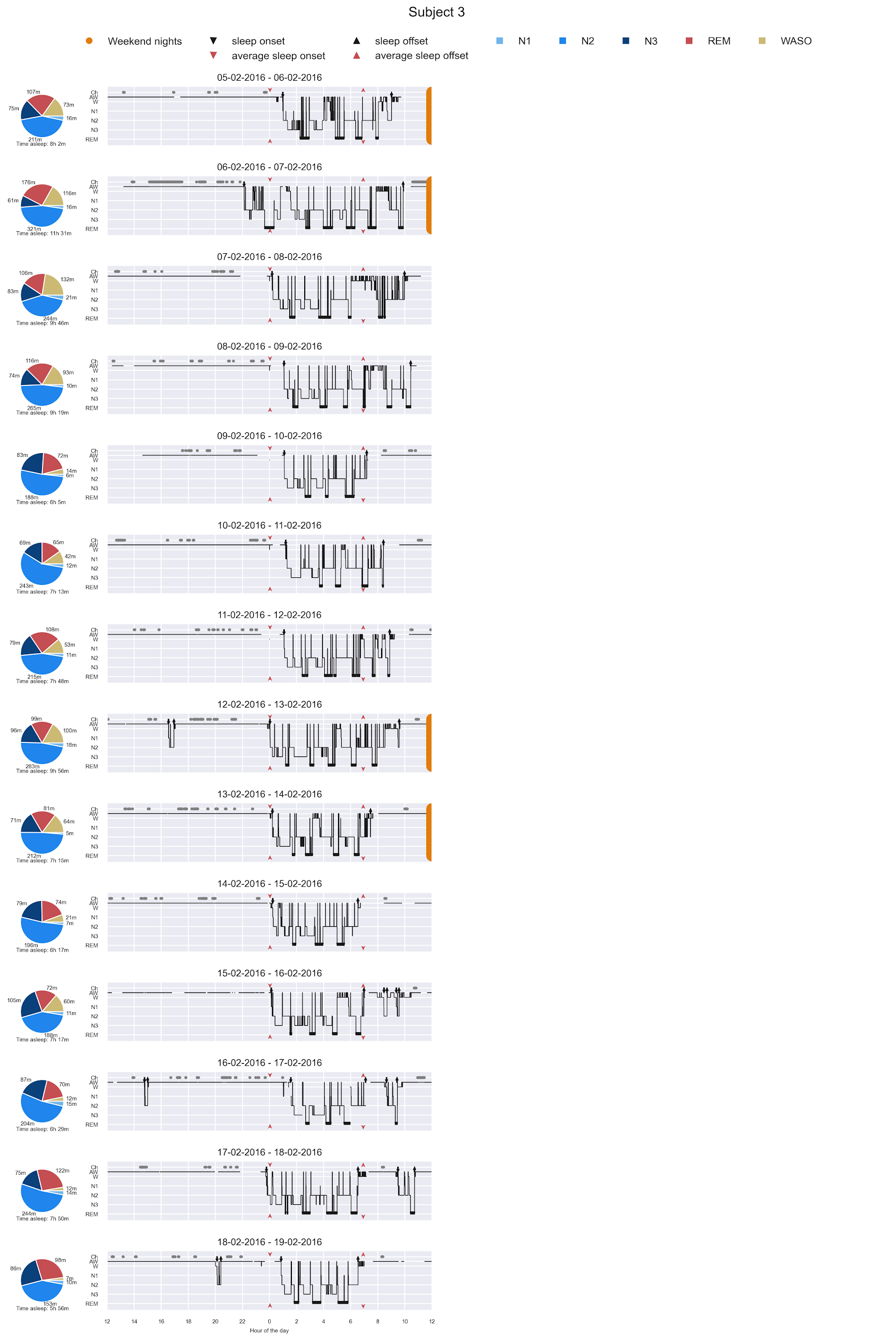
**

**
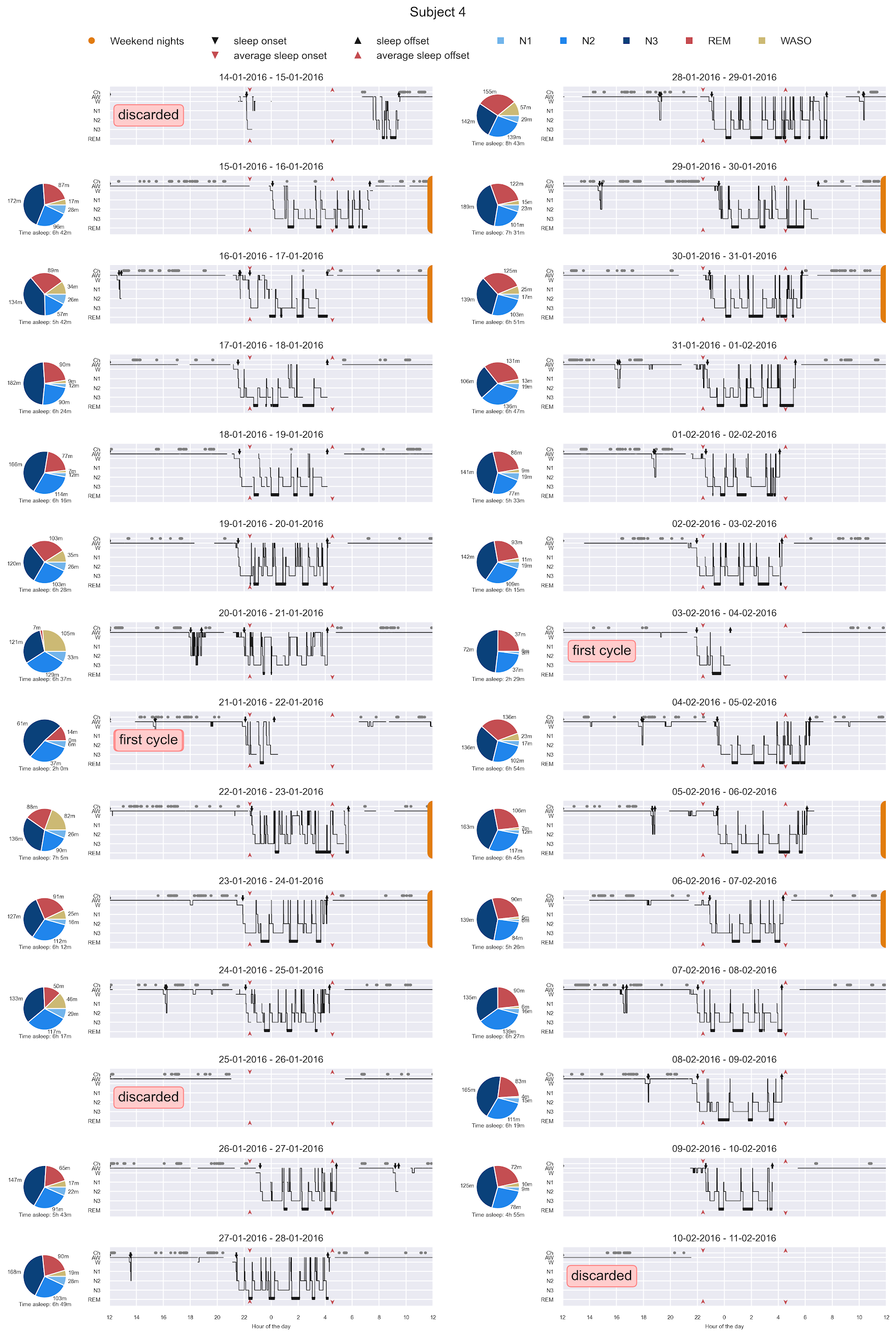
**

**
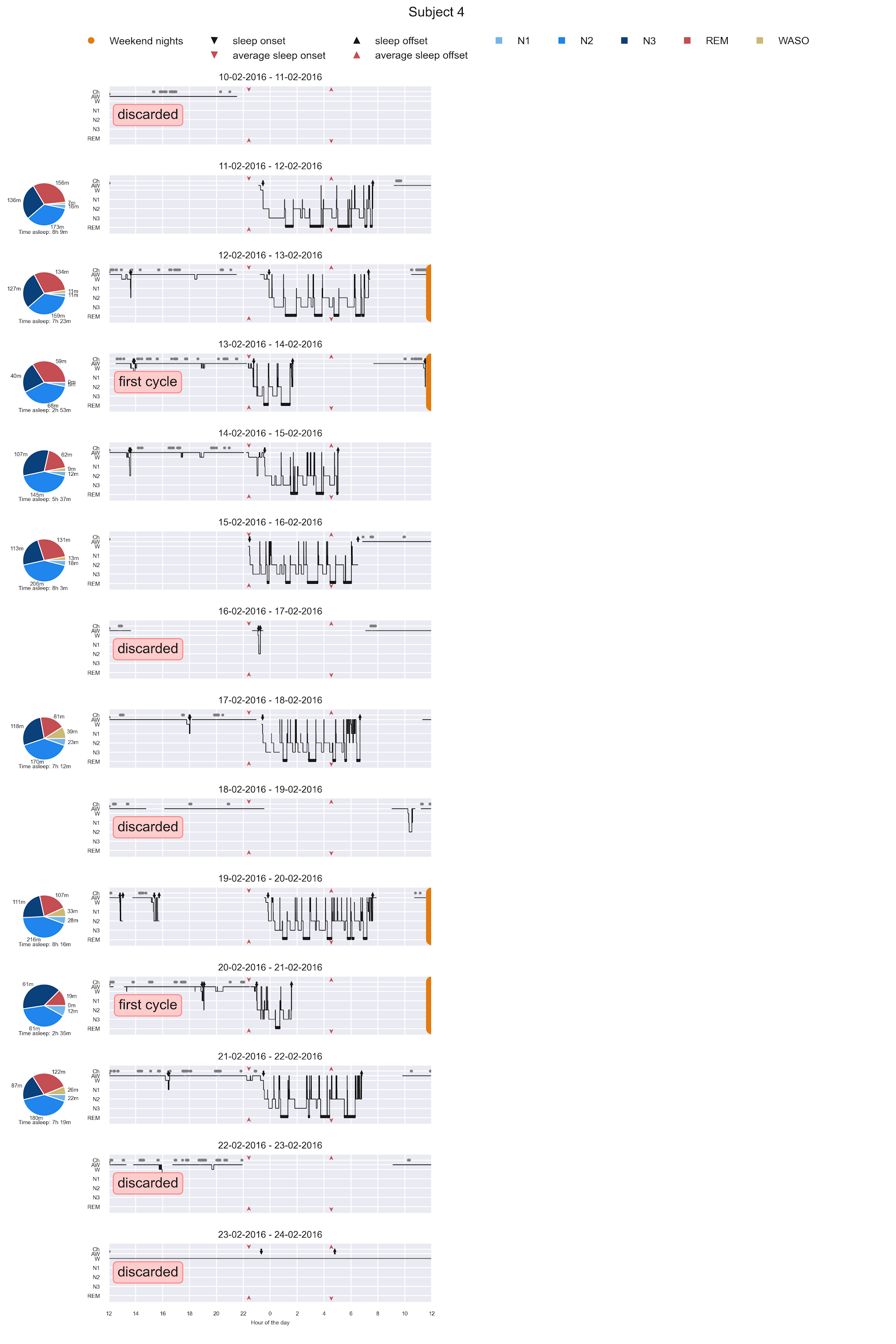
**

**
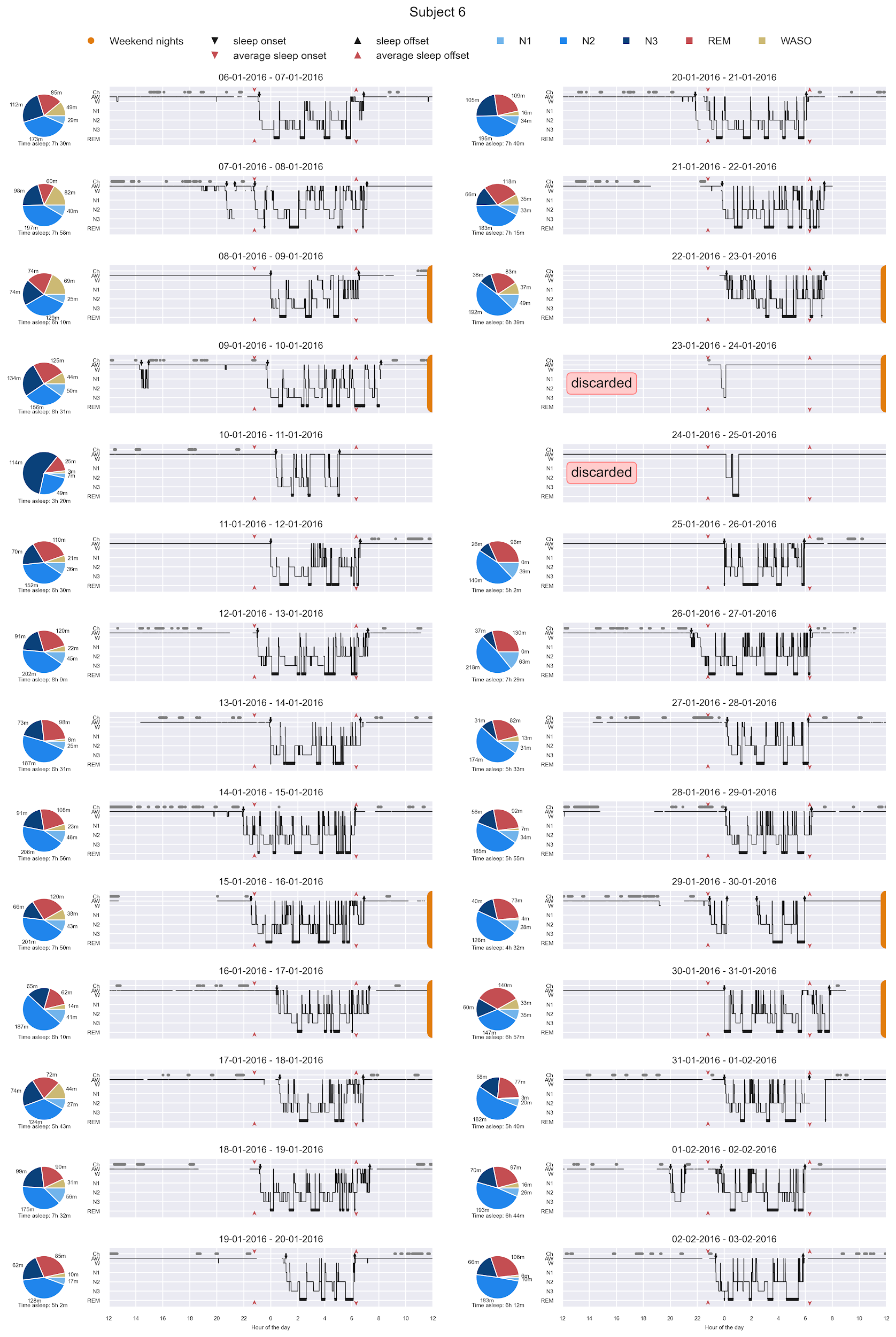
**

**
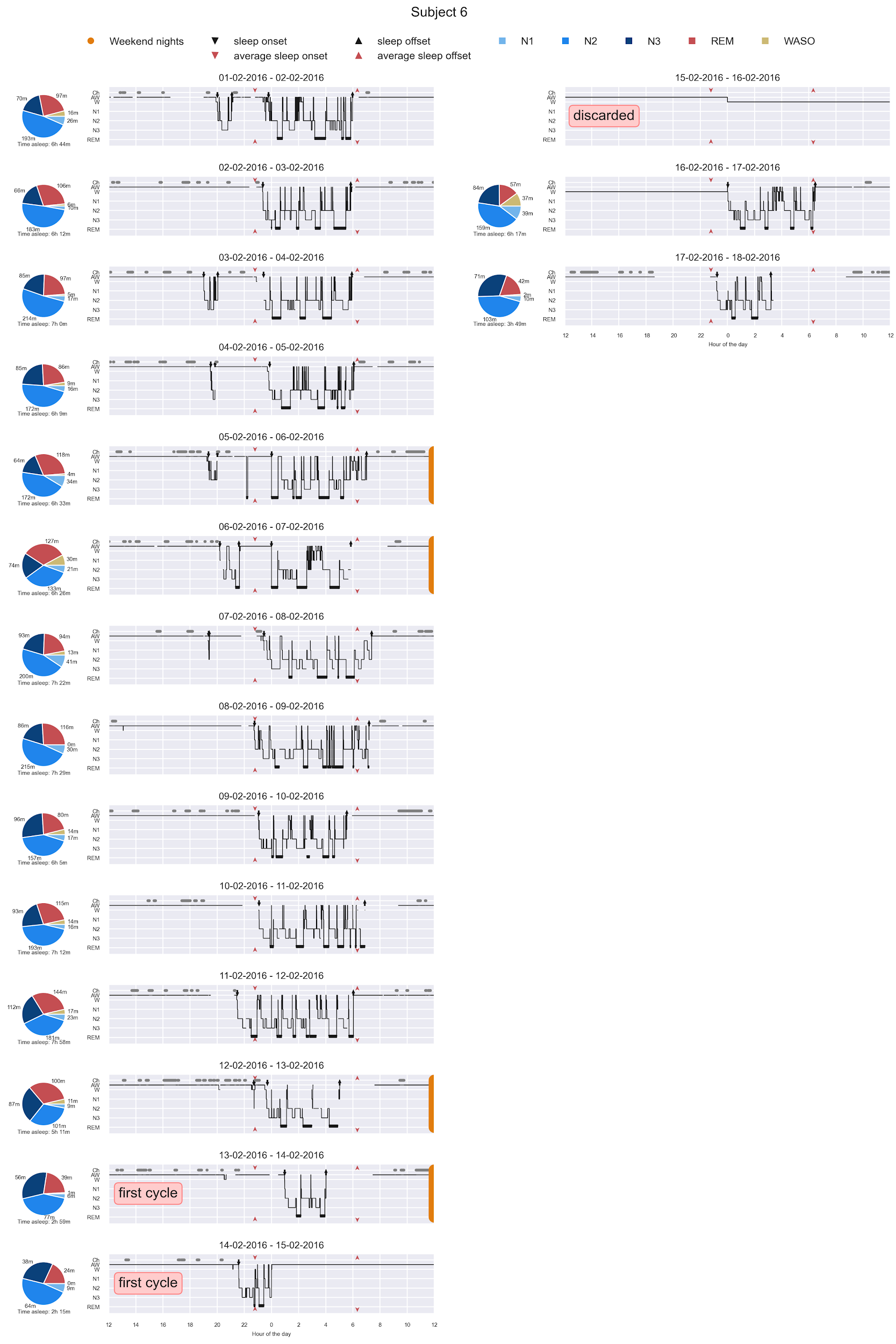
**

**
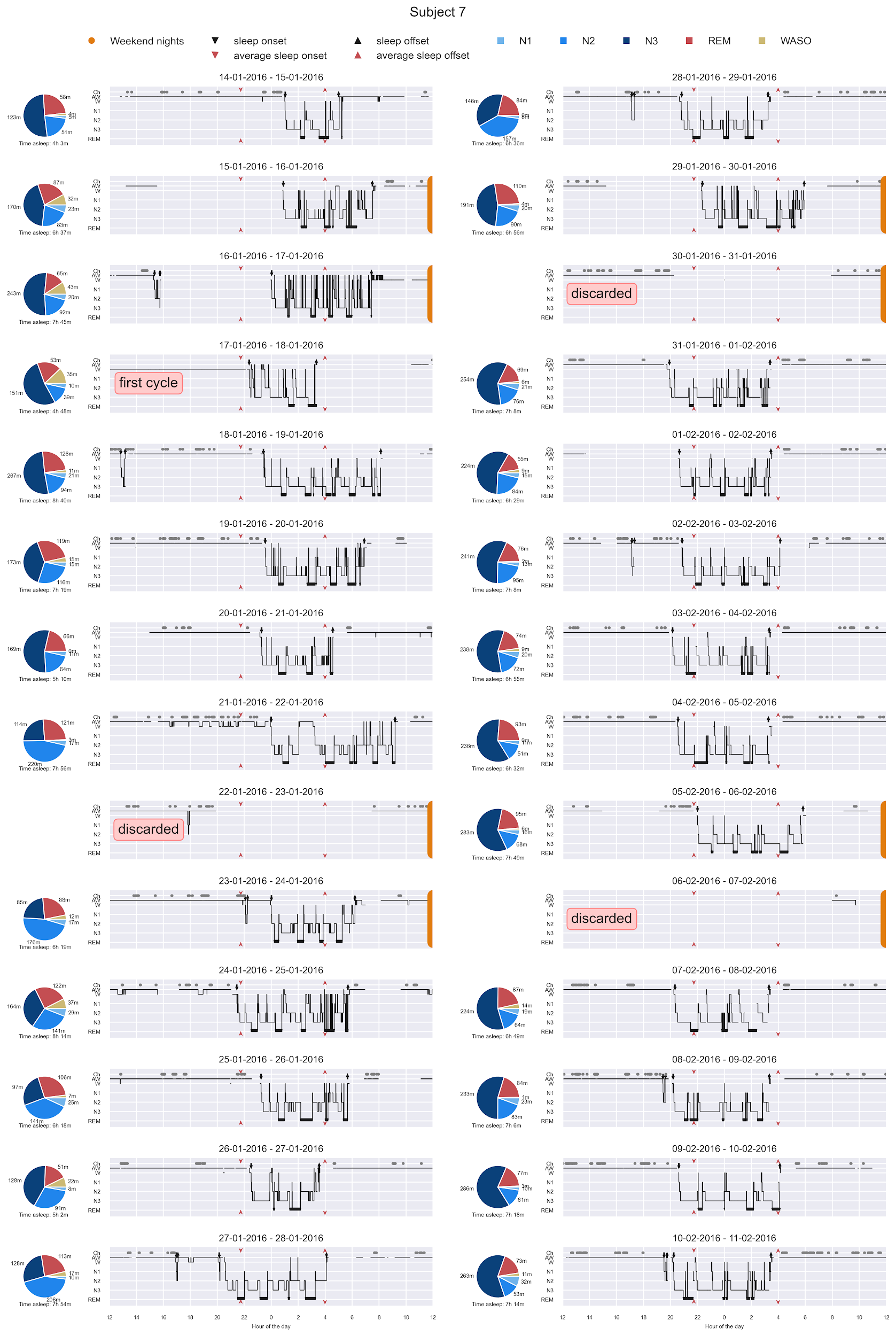
**

**
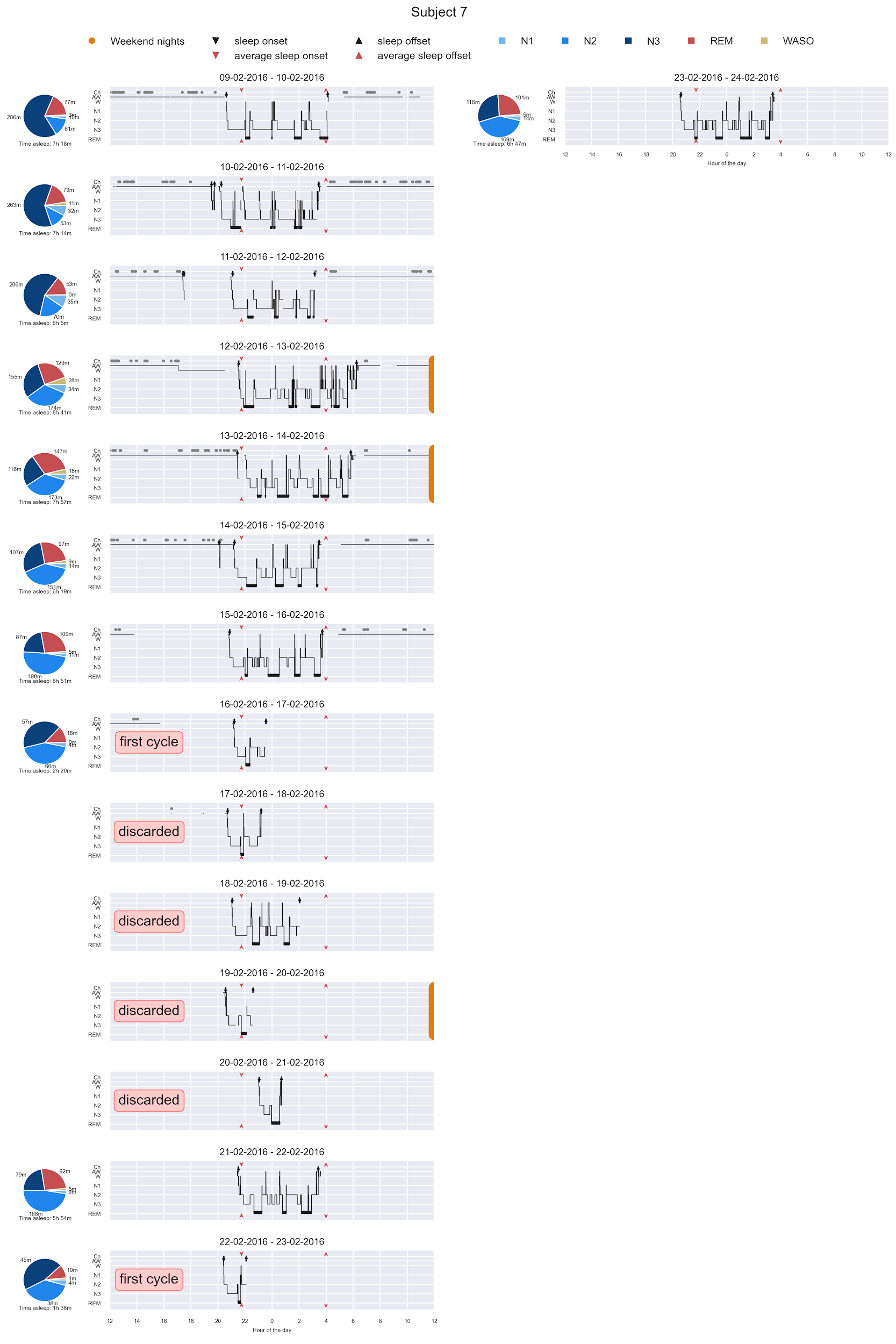
**

**
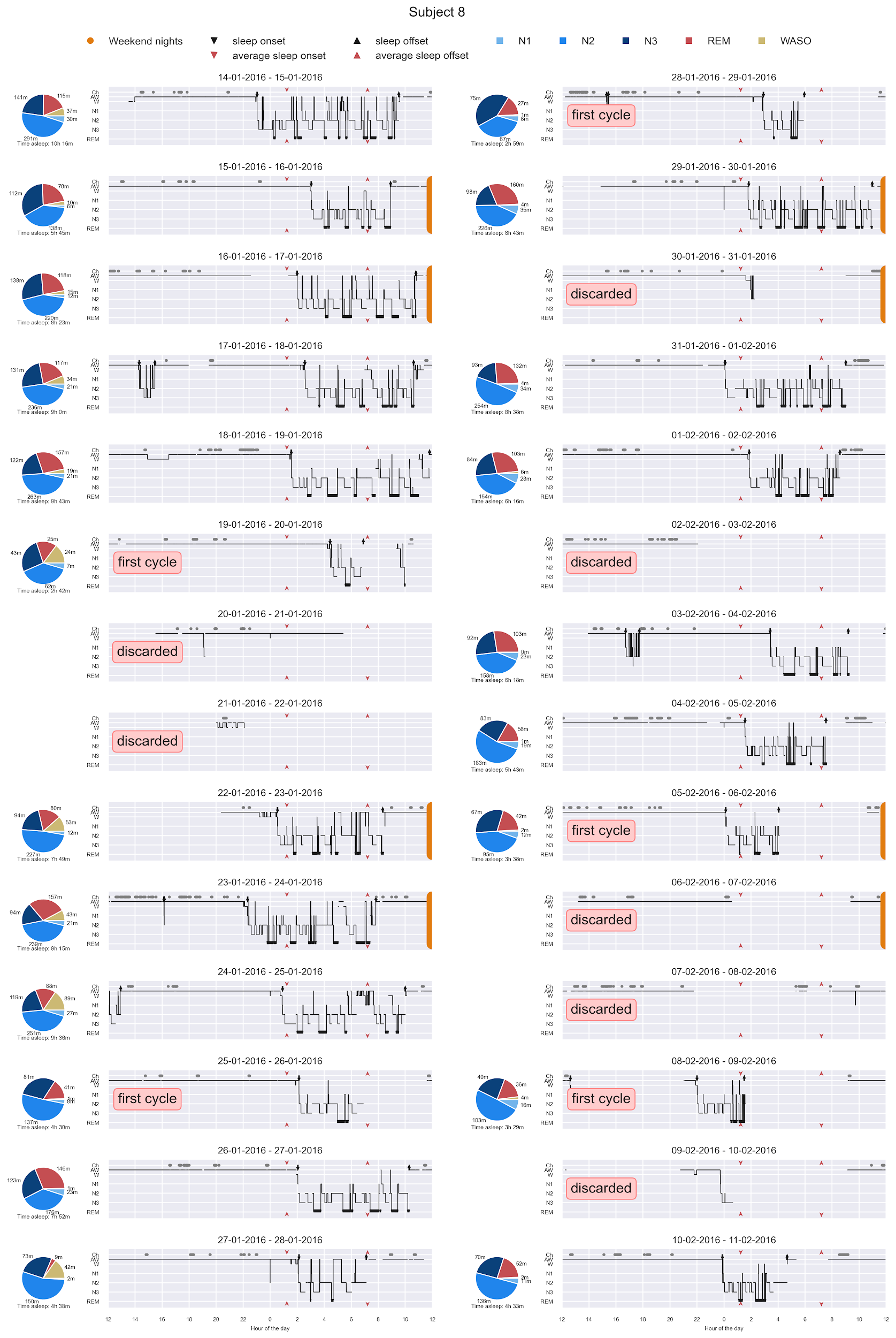
**

**
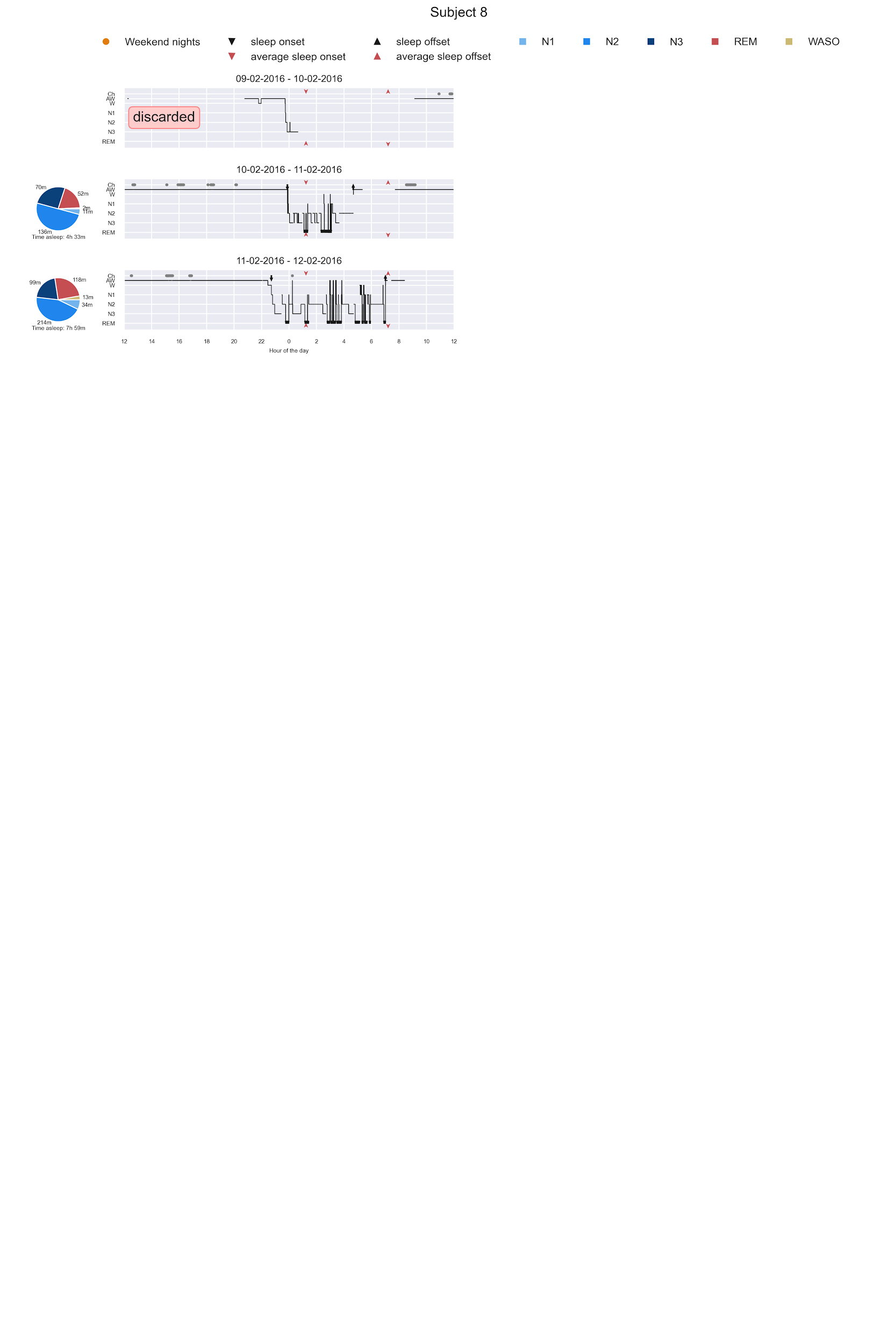
**

**
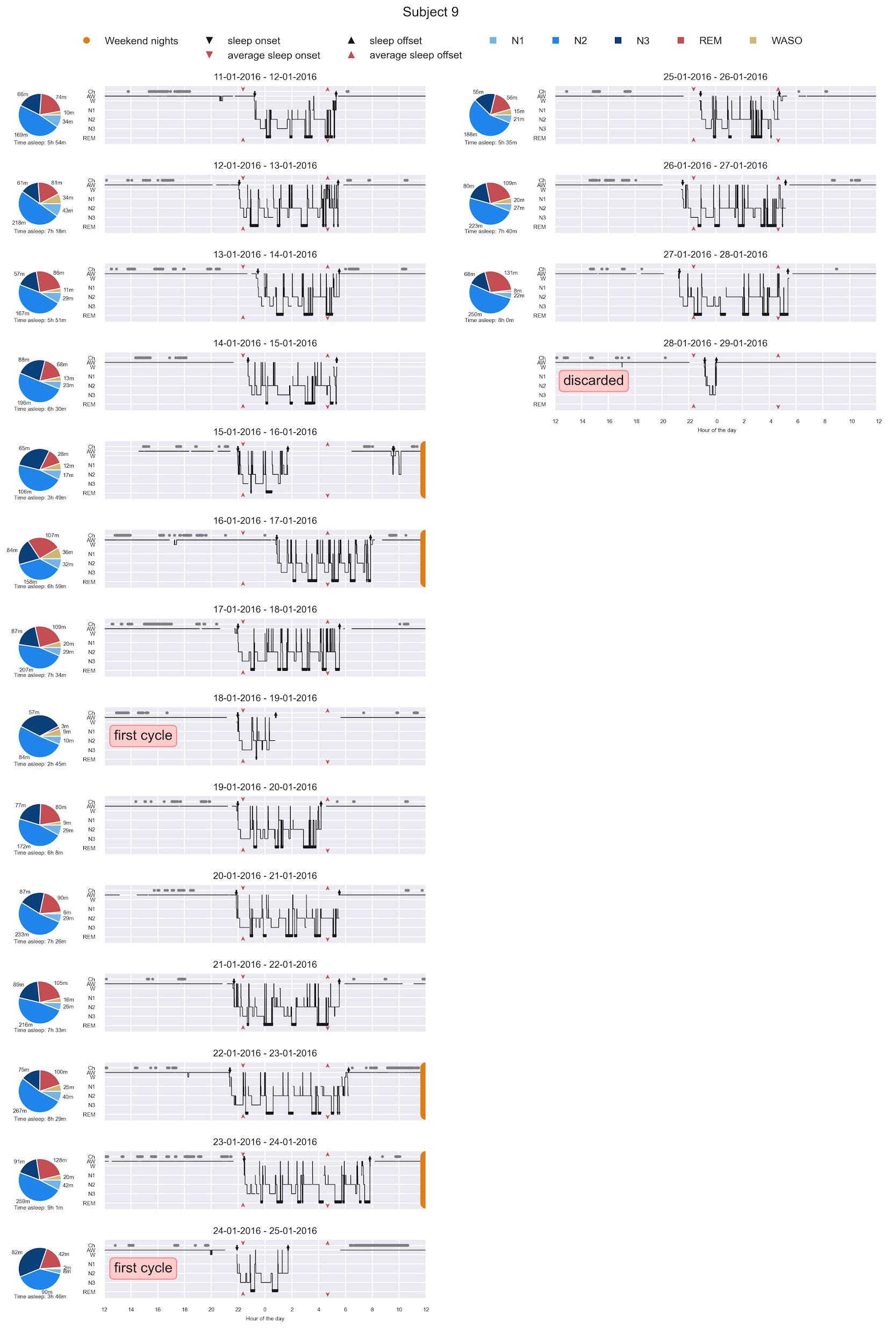
**

**
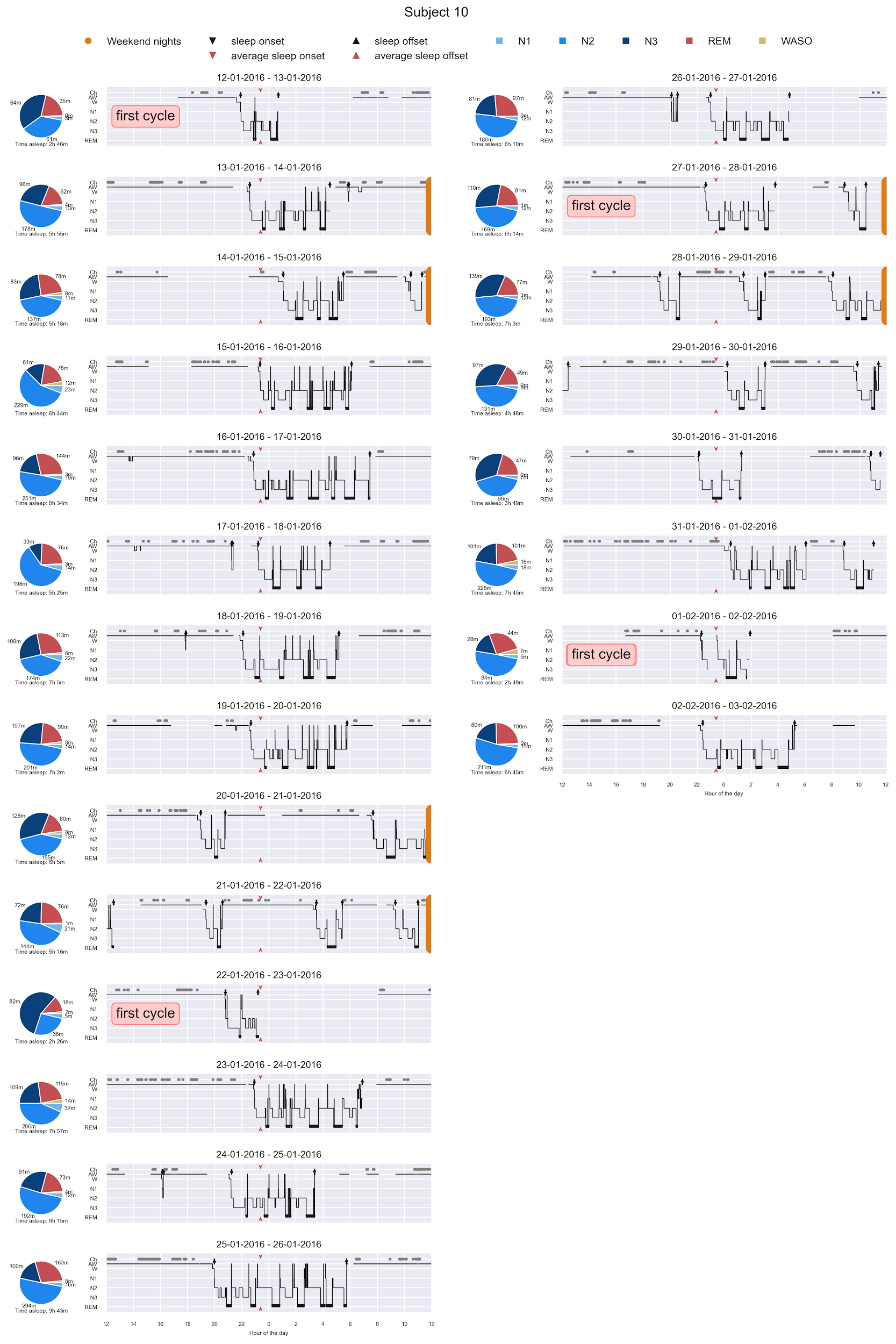
**

**Supplementary Figure 2: All scored data.** Raw scoring for all the nights scored from all subjects included in this study. For each plot, the score (y-axis) and the time of the day in hours (x-axis) are shown. Each night includes up (down) black arrows depicting the start (end) of the sleep and up (down) red arrows depicting the average start(end) of that subject. Additionally, information regarding the sleep stages distribution of that particular night is depicted as a pie chart with blue, red, and yellow portions corresponding to non-rapid eye movement (NREM) sleep, rapid-eye movement (REM), and wakefulness after sleep onset (WASO), respectively. Weekend nights are shown as orange bars at the left part of the plot. Interruption in the recording was handled as follows; Discarded: indicate those nights where data was insufficient for any analysis. First cycle: indicate those nights where only the first sleep cycle could be accounted for.

## Archetypal sleep patterns

Extracting an average sequence from a set of individual examples is a non-trivial issue, as the examples may not be aligned in time. We used an extension of DTW called dynamical time warping barycenter averaging (DBA) to extract an ‘archetypal sleep patterns’ for each subject by computing a representative night with lowest dissimilarity to all other nights. This representative average is useful in showing the central tendency of the individual sleep architecture and can also serve as a reference to calculate deviation from the mean, replacing pairwise comparisons.

**
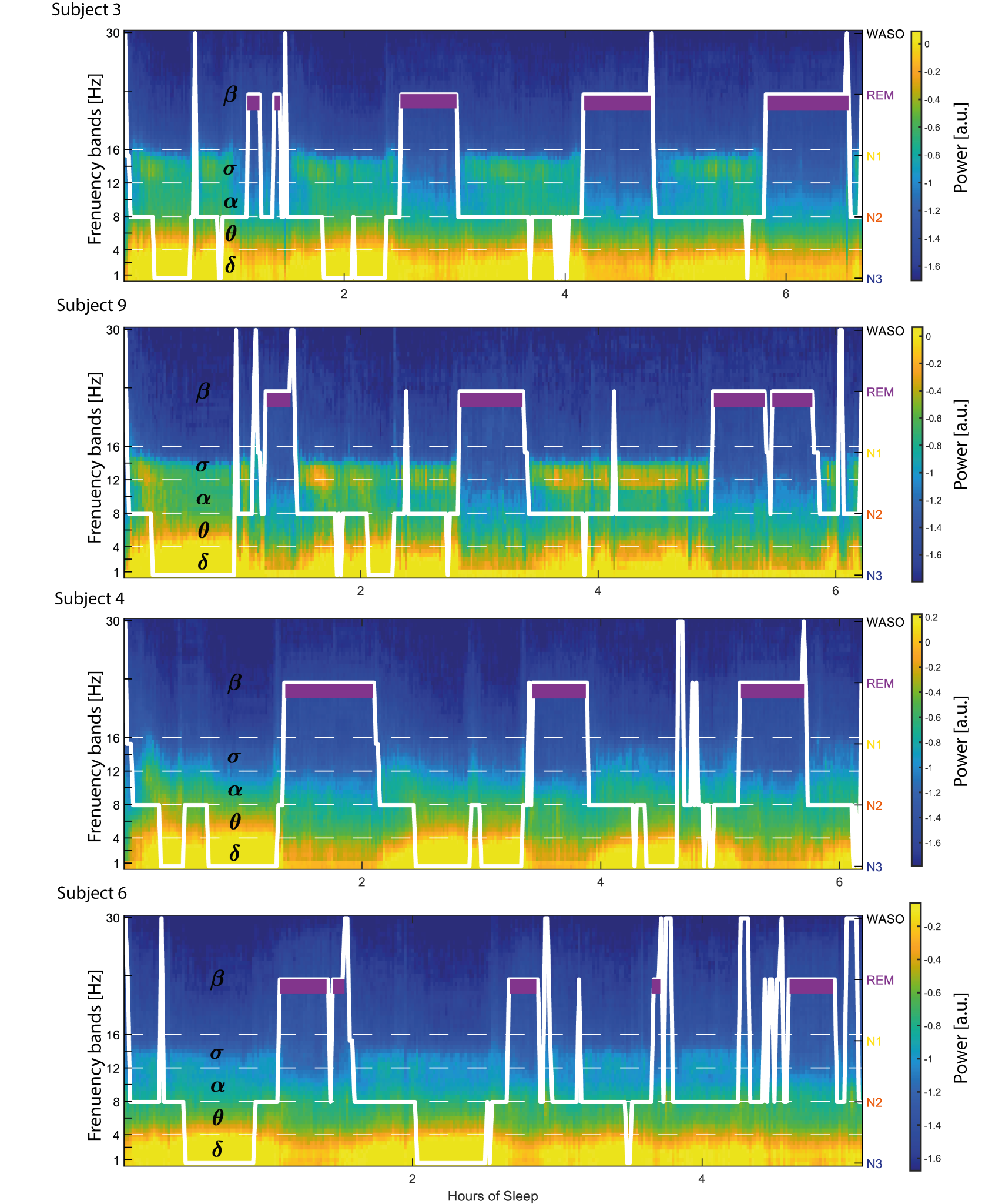
**

**
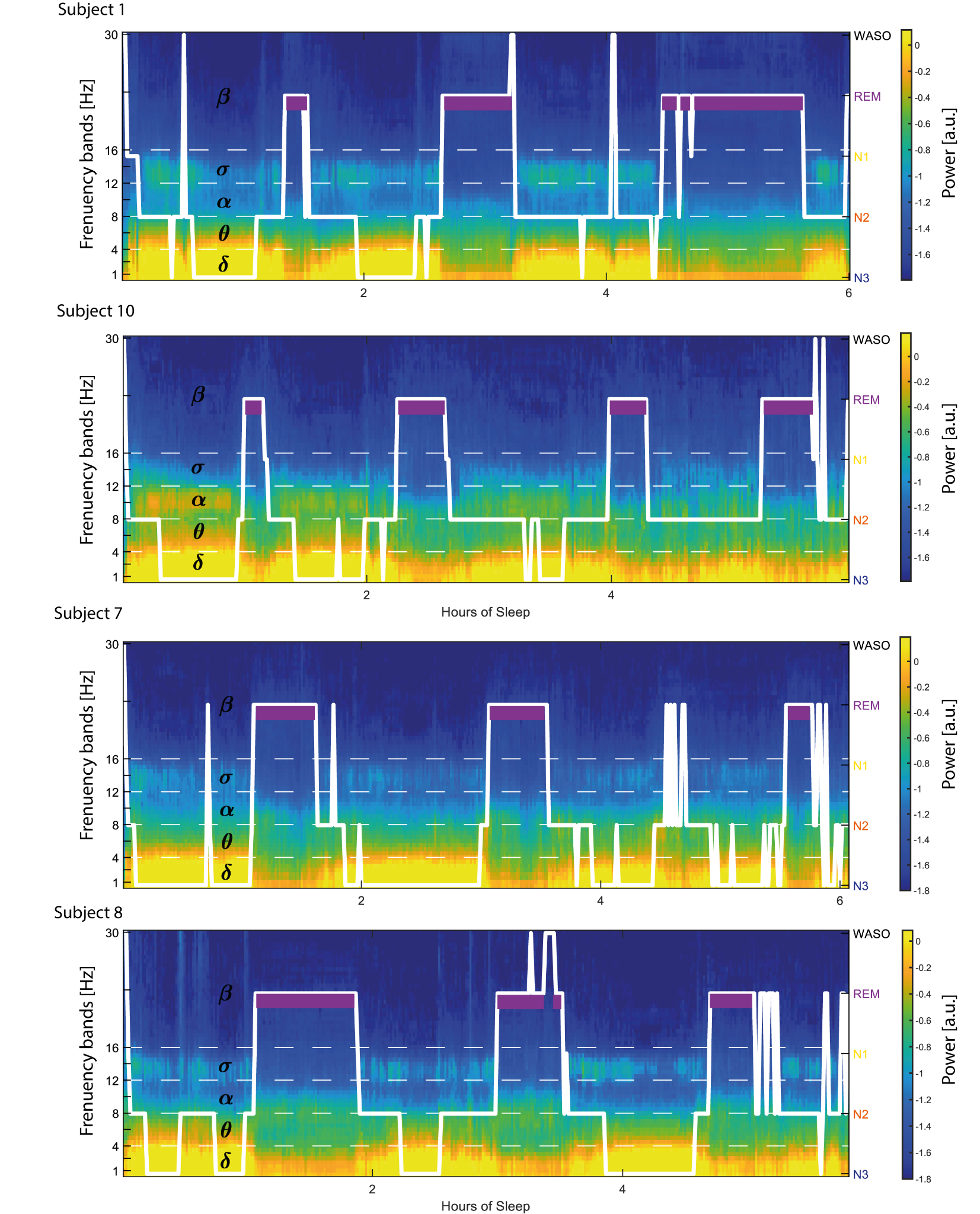
**

**Supplementary Fig. 3. Individual archetypal sleep patterns.** All individual centroids derived with the dynamical time warping barycenter averaging (DBA) algorithm. Average hypnograms are depicted with white traces and rapid-eye movement (REM) sleep with purple rectangles. Spectogram is normalized power with the following bands : δ[1-4Hz] , θ[4-8Hz], α[8-12Hz], σ[12-16Hz] ,β[16-30Hz].

**Deviations from the archetypal sleep pattern.**


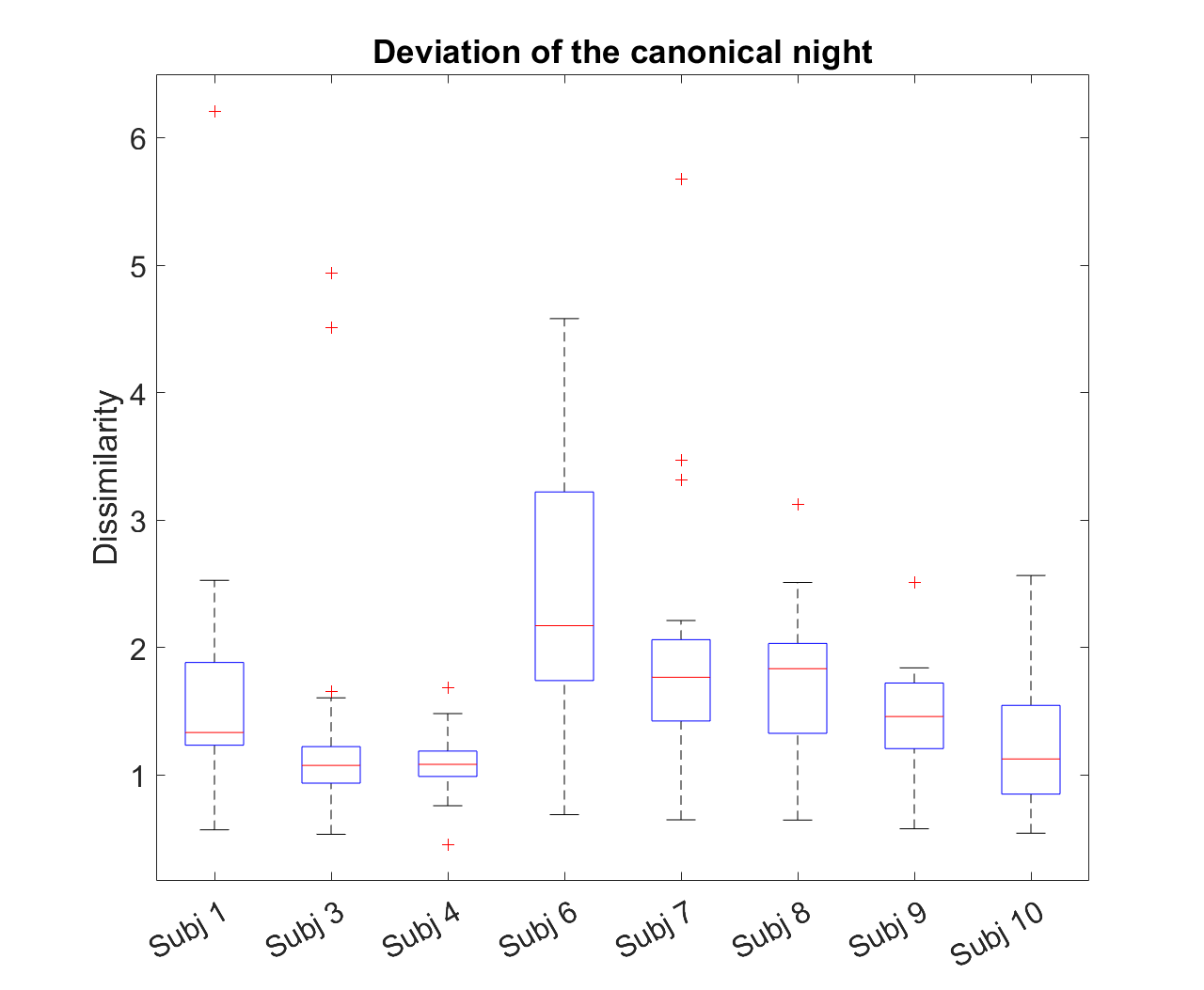


**Supplementary Figure 4. Average dissimilarity within subject.** Average and distribution of the dissimilarity index of individual nights compared to the subject-specific archetypal night as a reference. A higher average dissimilarity indicates more variable sleep deviating from the archetypal night. Subjects 3 and 4 present particularly consistent sleep. Subject 1, 7-10 have more variable sleep, whereas subject 6 has very variable sleep. Outlier nights of sleep that are highly dissimilar to the archetypal sleep pattern can be easily identified in almost all subjects (red pluses).

**Scoring individual nights of sleep based on the canonical night**

**
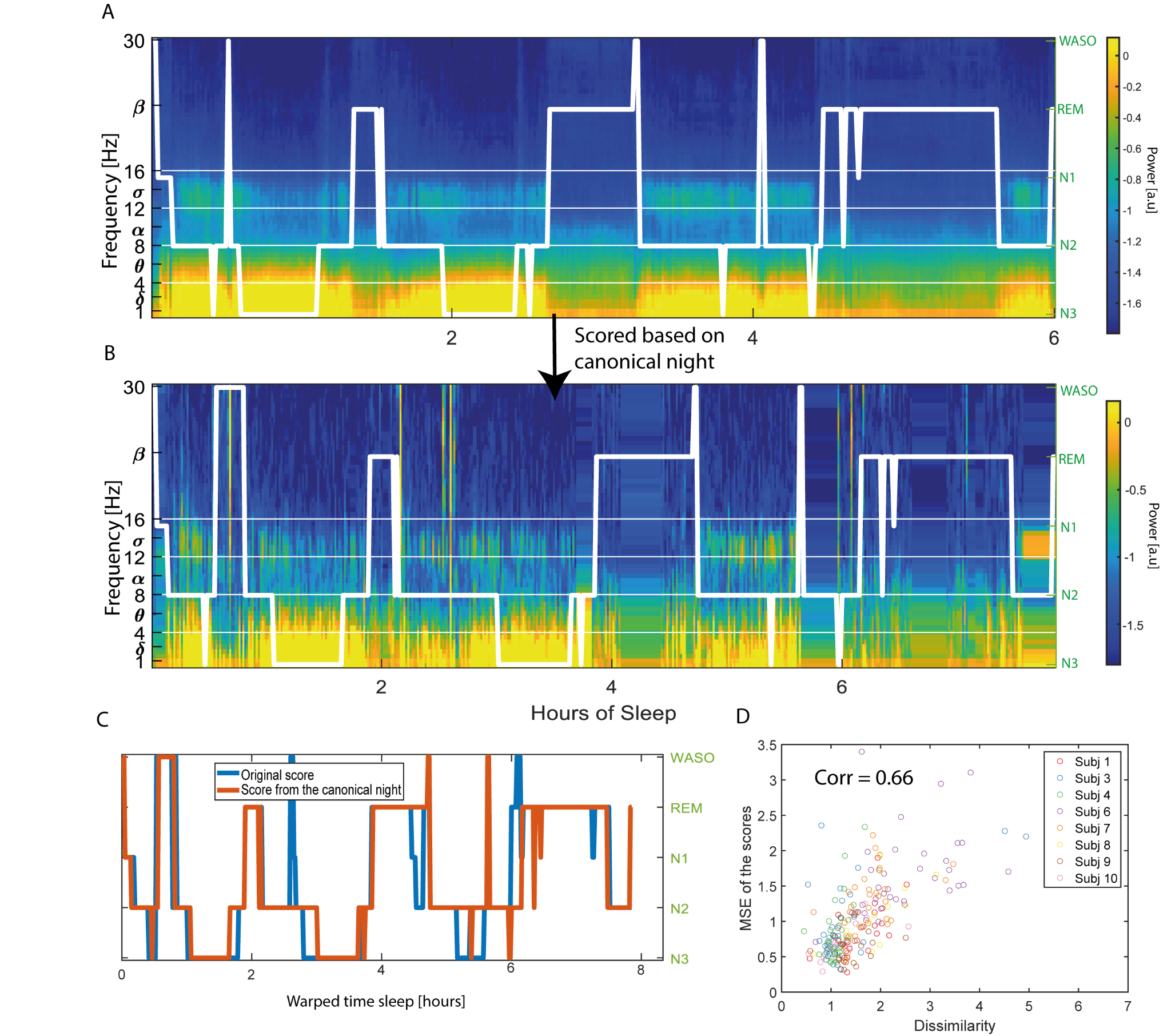
**

**Supplementary Figure 5. Scoring individual nights based on the canonical night.** (a) Spectrum of the canonical night drawn from subject 1 using open-ended dynamical time warping barycenter averaging (OE-DBA) (43 nights), along with a score obtained from the individual scores using the DBA indices. (b). Individual night 12th from the same subject warped to the canonical night using OE-DBA. Warping indices are derived from the 5-dimensional spectral warp, and applied on the individual spectrum and on the canonical sleep score (white trace). (c) Comparison between the original visual sleep score on night X and the score for night X derived from OE-DBA. (d) Relationship between the dissimilarity of the night to the canonical night and the error in scoring the night based on the mean squared error. The Pearson correlation coefficient was used to measure the linear correlation between the quantities in d.

**Comparison of static statistics and dynamic features**

To further characterize which factors influence our dissimilarity measure derived by OE-DTW, we used a correlative approach (Supplementary Fig. 6A). Significantly correlated factors are depicted in blue in Supplementary Fig.6B, along with the percentage of variance explained (non-significant in red). We did not find a single explanatory factor that explained a large portion of the variance in the dissimilarity between nights. We also found that several factors could have a weak contribution. Thus, the computed dissimilarity captures a summary statistic beyond those extracted through visual scoring.

Additionally, we also evaluated dissimilarity across sleep stages within the same subject. Indeed, OE-DTW is occasionally forced to align portions of the sleep-wake cycle that were independently scored as different stages by a visual scorer. On the rare occasions this occurred, dissimilarity was highest (e.g. REM-N3)., whereas it was lowest for what visual scorers considered being the same stage (Supplementary Fig. 6C, same as Fig.2D-E). **
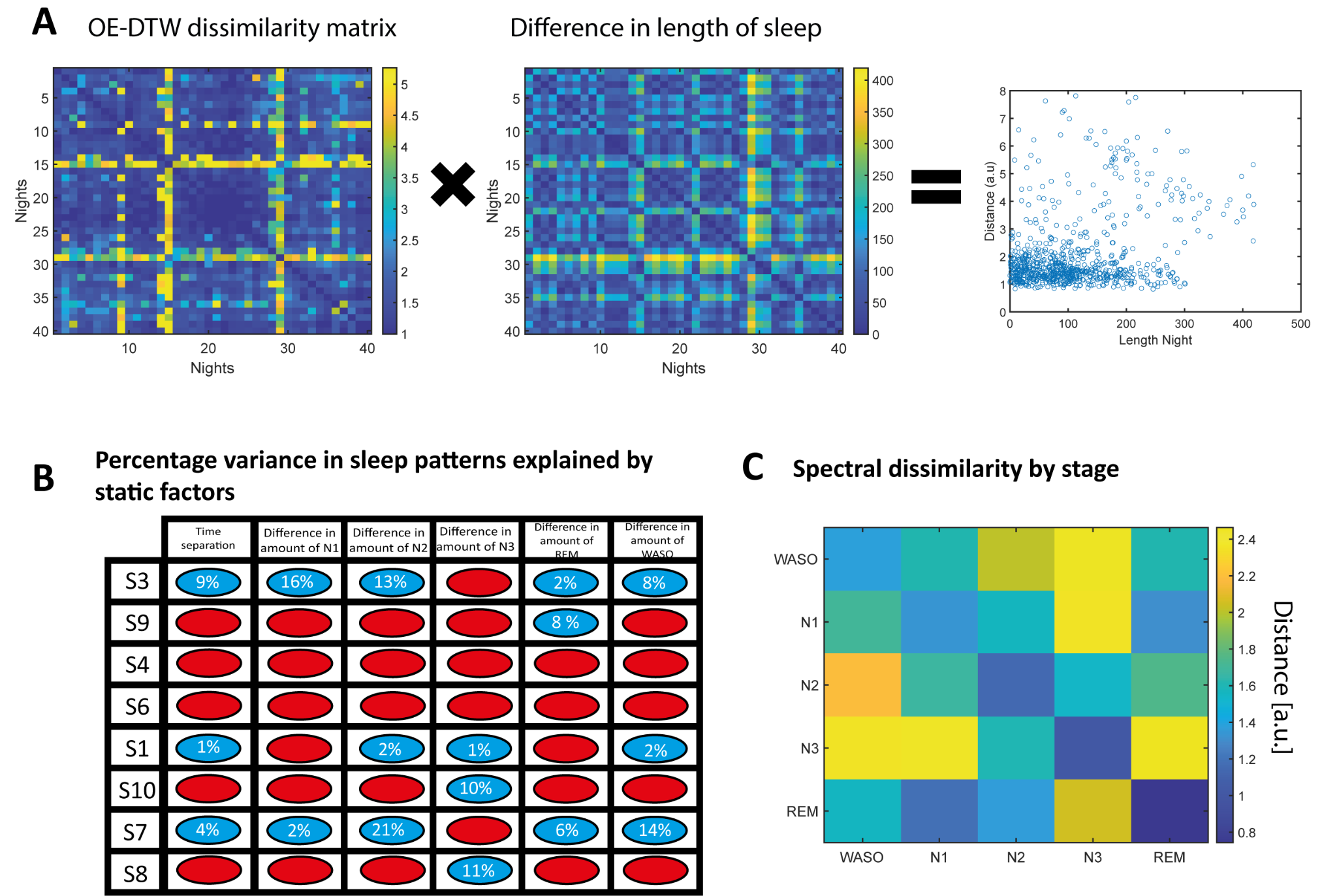
**

## **Supplementary Figure 6. Factors increasing sleep dissimilarity within subjects.** **A.** Example of one dissimilarity matrix in subject 1 (left) along with the difference of sleep length between all nights showing a weak, but statistically significant correlation. **B.** Table depicting all the significant (cyan) and non-significant (red) correlations between the pairwise dissimilarity between sleep recordings and a number of static factors. The percentage of dissimilarity explained by a given factor is indicated for significant correlations only (blue). The Pearson correlation was used to assess both the significance and the explained variance for all the quantities in table b. **C.** Dissimilarity matrix averaging all nights of all subjects. Diagonal elements correspond to average dissimilarity between the same sleep stages, whereas off-diagonal elements correspond to the dissimilarity when sleep stages are different between two nights.

**Sleep homeostasis and dissimilarity between nights**

Given that some dissimilarity stemmed from NREM, we next asked how much the expression of slow waves or spindles could explain this variability. Sleep homeostasis is known to affect the prevalence and amplitude of slow waves from one night of sleep to the next (increasing with prolonged time awake), as well as over successive sleep cycles (decreasing with time asleep, Supplementary Fig. 7A). Slow wave activity (SWA) can be measured during sleep as a marker of sleep homeostasis. Longitudinal EEG recordings open the possibility to track sleep homeostasis over many days in the every-day environment, beyond the controlled and artificial sleep manipulations, classically carried out in the setting of sleep research labs. As a ‘natural experiment’, we asked whether variations in sleep amount over time would result in variations in sleep homeostasis. As expected, we found that SWA upon falling asleep (at least to N2) during daytime (naps) or night-time increased logarithmically as a function of prior time spent awake (Supplementary Fig. 7B, left). Additionally, SWA decayed exponentially as a function of time spent asleep (SFig. 7B, right). The coefficients of the model td, ti are depicted in SFig 7D. We modeled the process *S* of sleep as previously done with two separate exponential functions, one describing the accumulation of sleep pressure while awake:

$S(t) = (S_{WU}-UA)e^{(-t/\tau_{i})}+UA$

the other describing the decrease of sleep pressure while sleeping:

$S(t) = (S_{SO}-LA)e^{(-t/\tau_{d})}-LA$

The time constants $\tau_{i}$ and $\tau_{d}$represent the increasing and decreasing sleep pressure *S* during wake and sleep, respectively. The variables $S_{WU}$ and $S_{SO}$ account for the sleep pressure at wake-up sleep onset. Finally, UA and LA stand for the upper and lower asymptote.

We also checked whether sleep homeostasis affects the similarity measure. To do that, for each subject, we compute the difference of single nights to their canonical one as a function of the median SWA/N3. We observe that the more deviation the SWA and N3 from the median of the subject, the more dissimilar the nights are (sFig. 7c). This effect seems to be quite symmetrical, showing that sleeping more than average or less than average affects in a similar way to the overall architecture of the night. This homeostatic effect is not replicated if instead of N3 we use REM. Thus slow waves can vary between nights and our dissimilarity metric can help identify nights with more SWA


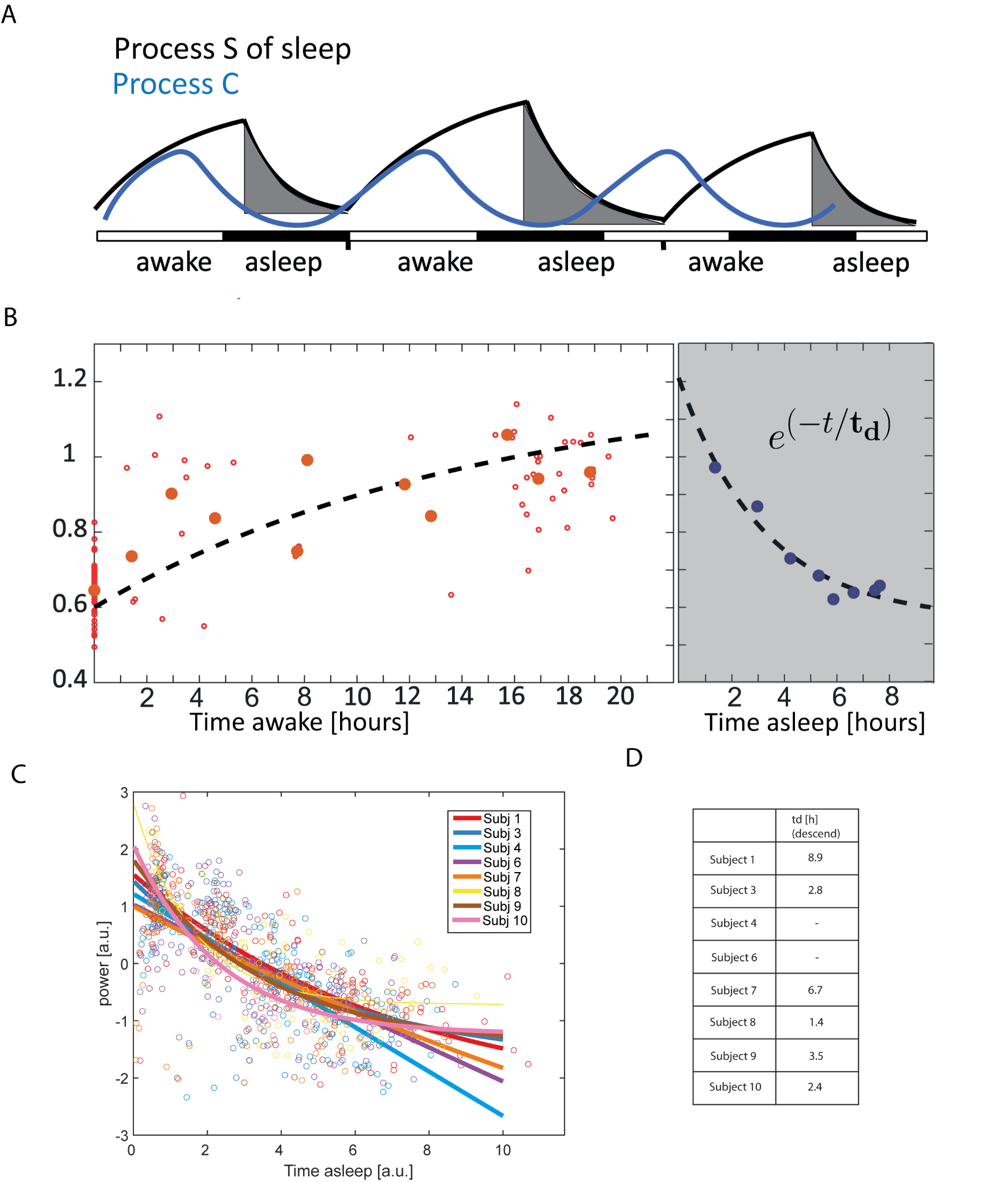


**Supplementary Figure 7.** Sleep homeostasis over the sleep-wake cycle and across nights within subjects. **A**. Schematic representation of the two-process model of sleep depending on Process S (Sleep homeostasis) and Process C (Circadian rhythm). **B.** Mean SWA activity as a function of the time awake (red) or asleep (blue) across days and nights recorded from one subject. SWA was also computed for daytime sleep (i.e. naps) if the subject reached at least N2. The available data was binned into one-hour bins during the daytime. **C.** One SWA exponential decay fit per subject as a function of the time sleeping. **D** Table with the decreasing and increasing coefficients of the exponential model td.

**
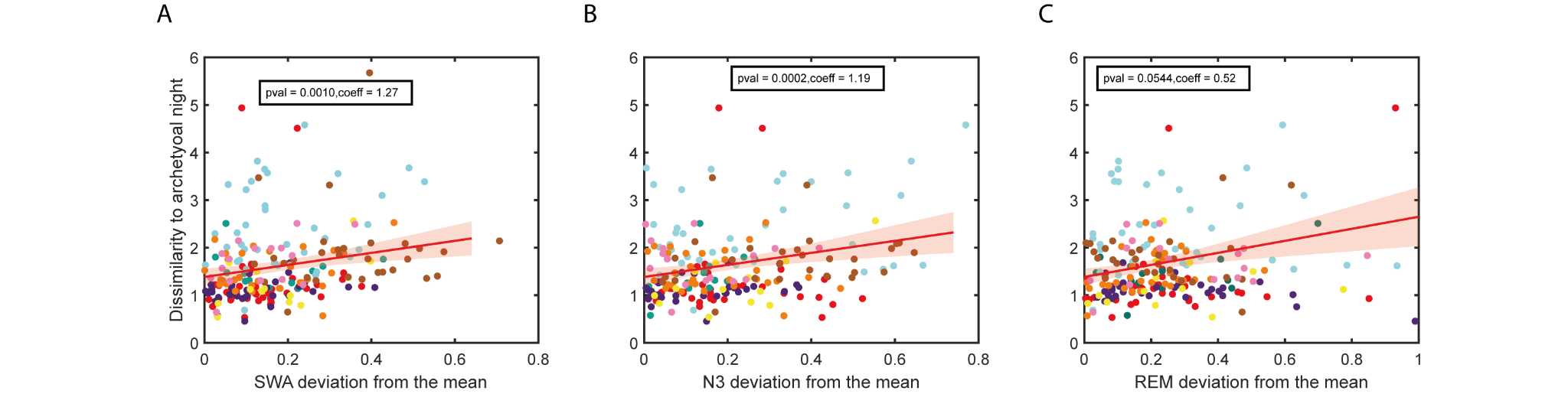
**

**Supplementary Figure 8. Correlation between dissimilarity and sleep homeostasis.** Dissimilarity to the subject-specific archetypal night as a function of the difference to the archetypal SWA (A), N3 sleep duration (B) and REM sleep duration (C). The p-values are derived from the significance of a linear regression model attempting to model the dissimilarity using the three variables separately (SWA, N3, and REM). The red line and red shadow represent the linear model and 95% confidence interval. Patient colors are taken from SFig 7.


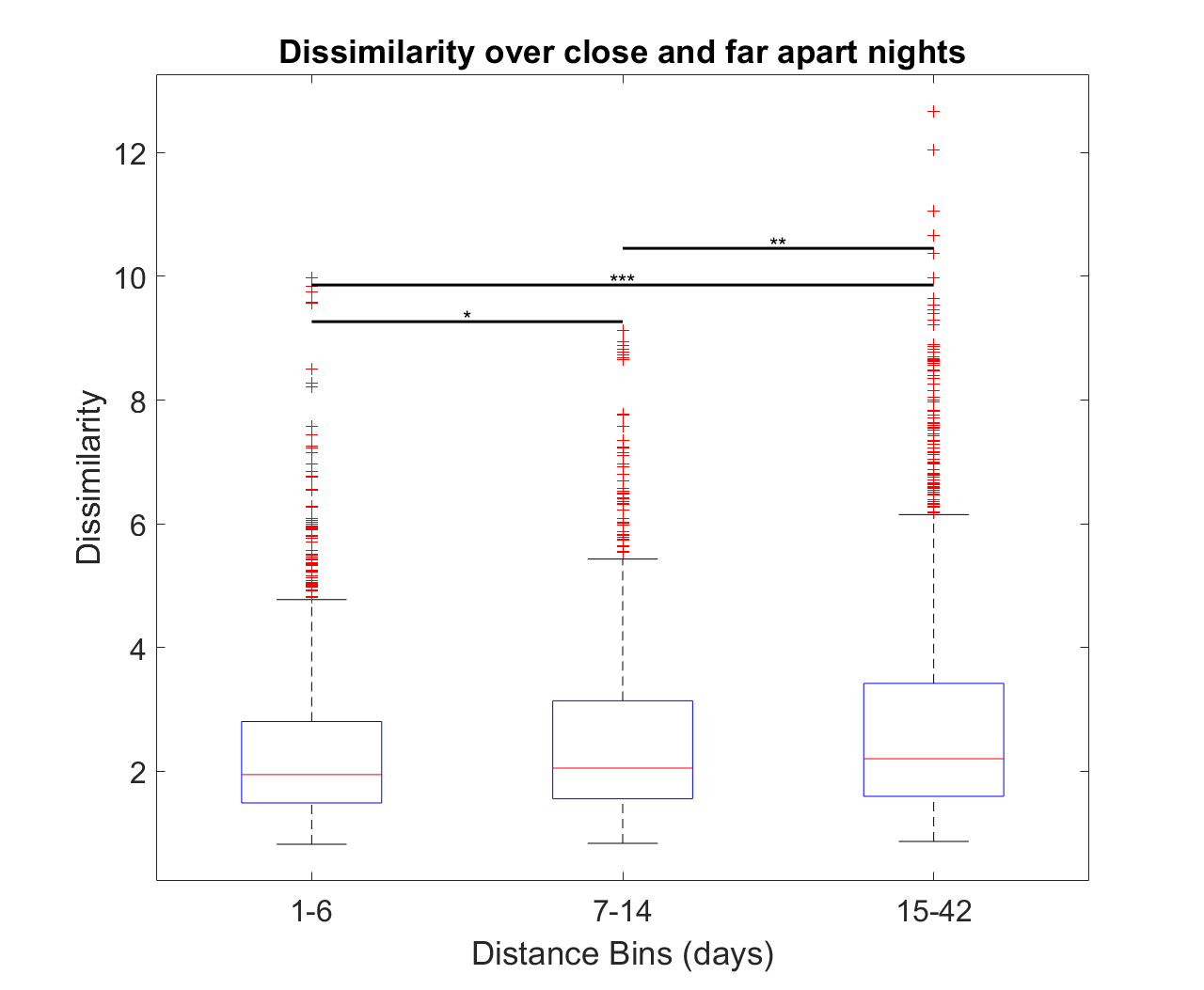
**Supplementary Figure 9. Separation interval and dissimilarity.** Consecutive nights within a week are more similar than pairs of nights separated by longer intervals of one or more weeks. DTW dissimilarity pooled across subjects and binned by separation interval in days. The Kruskal-Wallis test was applied to test if the three boxplots come from the same distribution (0.05 *, 0.01 **, 0.001 ***).

##


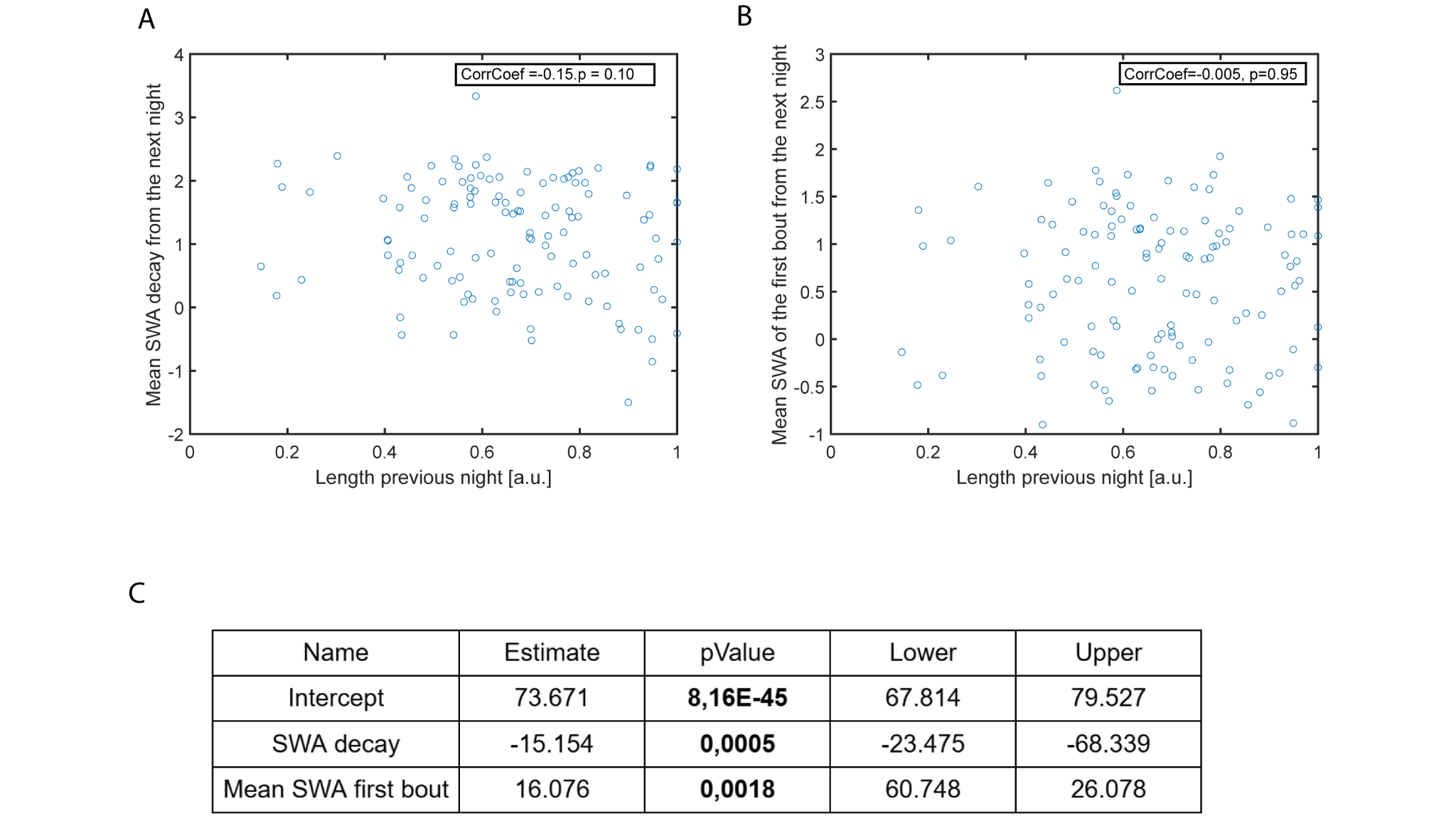


## **Supplementary Figure 10.** A. Sleep homeostasis is defined by the comparison of the length of the previous night to the difference between the first and last bout of the next night. B. Same as in A, but from the mean SWA from the first bout. The Pearson correlation coefficient was used to measure the linear correlation between the quantities in A and B and to compute their p-values. C. Linear mixed model using the two variables from A,B with the subject id as a random effect. Significant variables are shown in bold in the pValue column.

**Slow-wave detection**

We filtered the data between 0.5 and 2 Hz (orange line in supplementary Fig 11a). We adapted the slow wave algorithm to be applicable to our data. We filter the data (previously scored as N2 or N3) between 0.2 and 2 Hz and annotate the positive and negative peaks along with the mid-crossing of the filtered data. We only select positive and negative peaks with a range of positive and negative mV. For technical reasons, we do not have a reliable measure of the exact microvolts of the reading of the electrodes, and adopted an amplitude threshold to detect the peaks of the slow waves consistently against our visual inspection. For each negative peak, we find the next positive peak and make logical thresholds such as duration, amplitude etc to find the final SW candidates. The candidates of the start and end of the slow waves are depicted in black and red, respectively. Additionally, we showed the average length of Slow-wave (Fig. 11b) and its typical wave-form (Fig.11c).


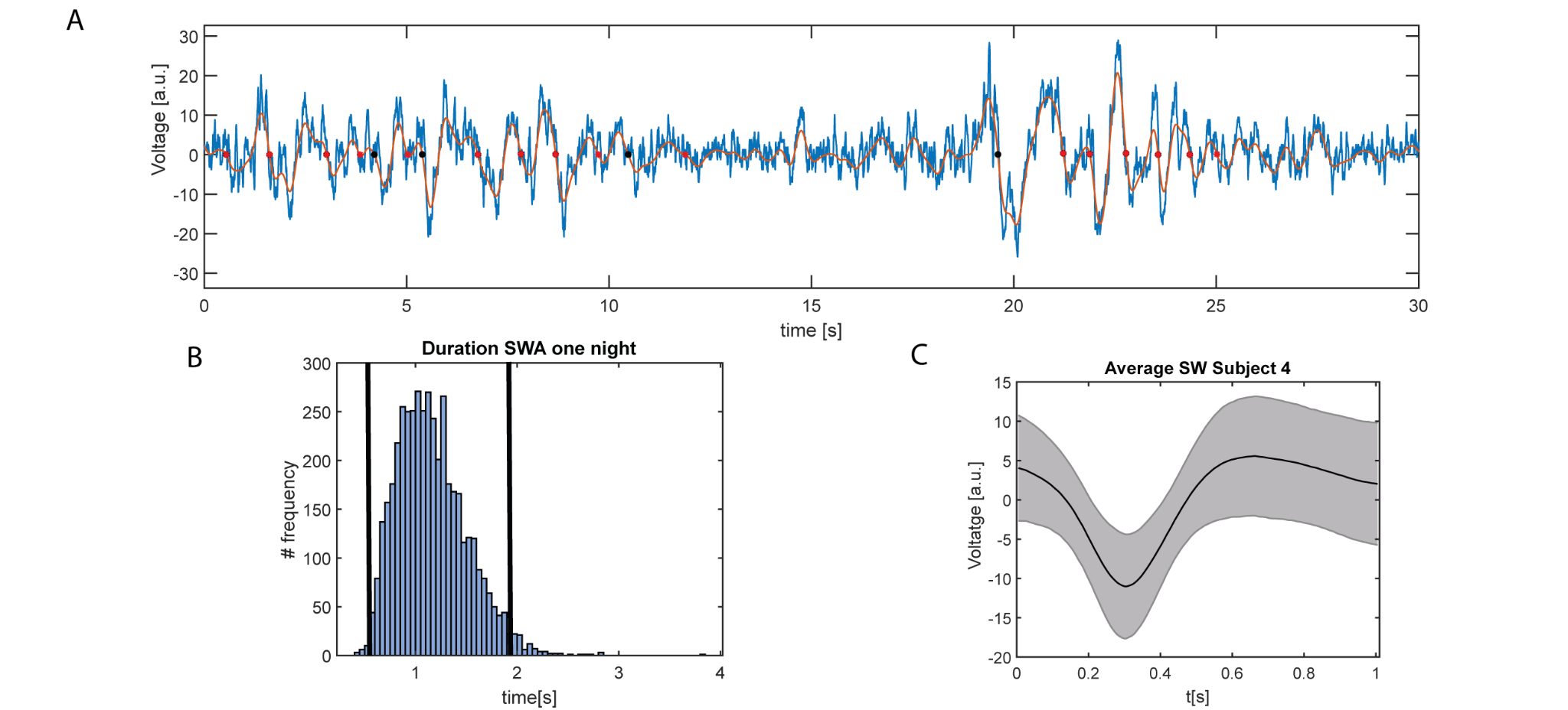


**Supplementary Figure 11. Slow wave detector.** a) Trace example of the subscalp electroencephalographic (sqEEG) for 30s along with the filtered signal between 0.5-2Hz. Black (red) dots indicate the initial (final) points of the candidates for the Slow-Waves (SW). b) Histogram of the duration of the SW for the computation of a single night. Black bars represent the threshold for two small or too big SW. c) Average SW for a subject with the classical shape of the SW.

Similarly, we plotted the filter signal between 11-16Hz for the spindl detector (Fig. 12). We also show four candidates with dots and the final selected in orange.


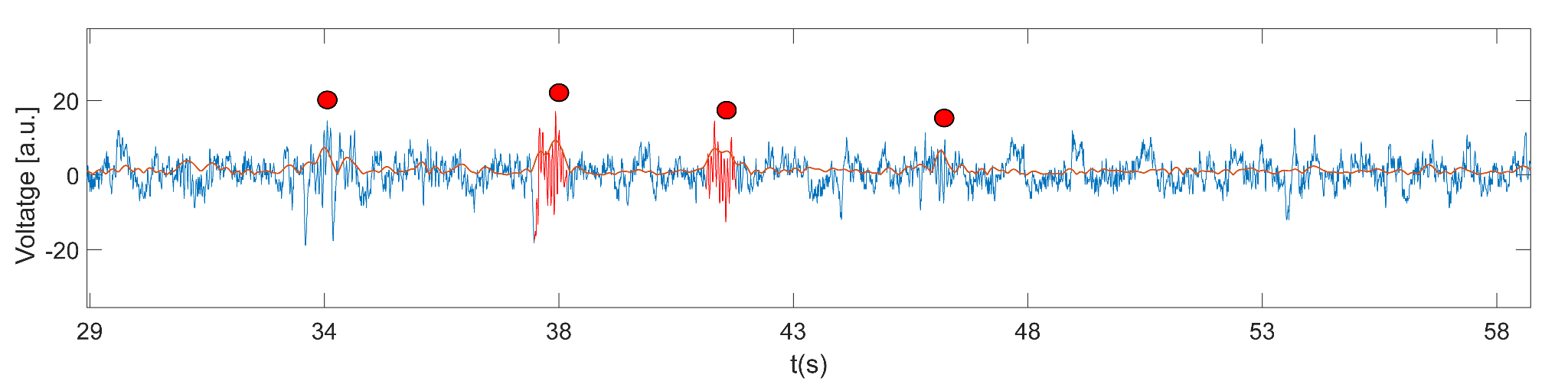


**Supplementary Figure 12. Spindl detector.** a) Trace example of the subscalp electroencephalographic (sqEEG) for 30s along with the filtered signal between 11-16Hz. Red dots indicate the initial candidates for the Spindles and the red sqEEG signal indicate the final Spindl detection after the thresholding.


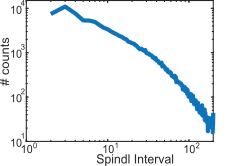


**Supplementary Figure 13. Infra-Slow variation of Spindel.** a) Number of Spindel occurrences as a function of their interval.


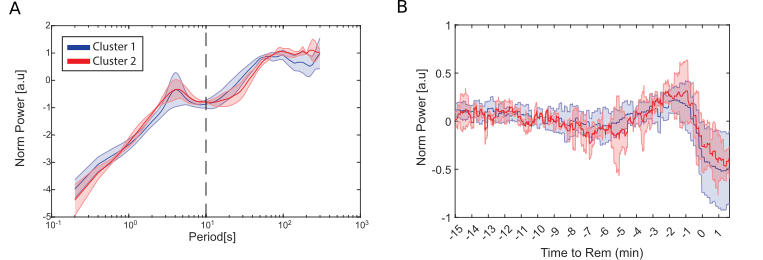


**Supplementary Figure 14. Infra-Slow variation of Spindl power per cluster.** a) Infraslow oscillation of the sigma power [12-16Hz] for the two clusters of subjects. b) Spindl power before rapid-eye movement (REM) sleep for the two clusters of subjects.

**Circular statistics:** We used circular statistics to assess the locking between the phase of a rhythm (Slow waves) and an event (Spindl occurrences). We first filter the signal between 0.5 and 2 Hz and then apply a Hilbert transform to compute the phase of the Slow waves. Then for each Spindl we take the phase of the slow wave a the Spindl centre (𝝋_j_) and compute:

$$Re^{i\psi}=\frac{1}{N}\sum_{j=1}^{N} e^{i\varphi_{j}}$$

where R is the mean phase-locking value (from now on PLV) and 𝝍 is the mean phase. The R value gives an idea of the synchrony between the two events. A value of R=1 is for a perfect locking whereas R=0 when the two events are completely unsynchronized.


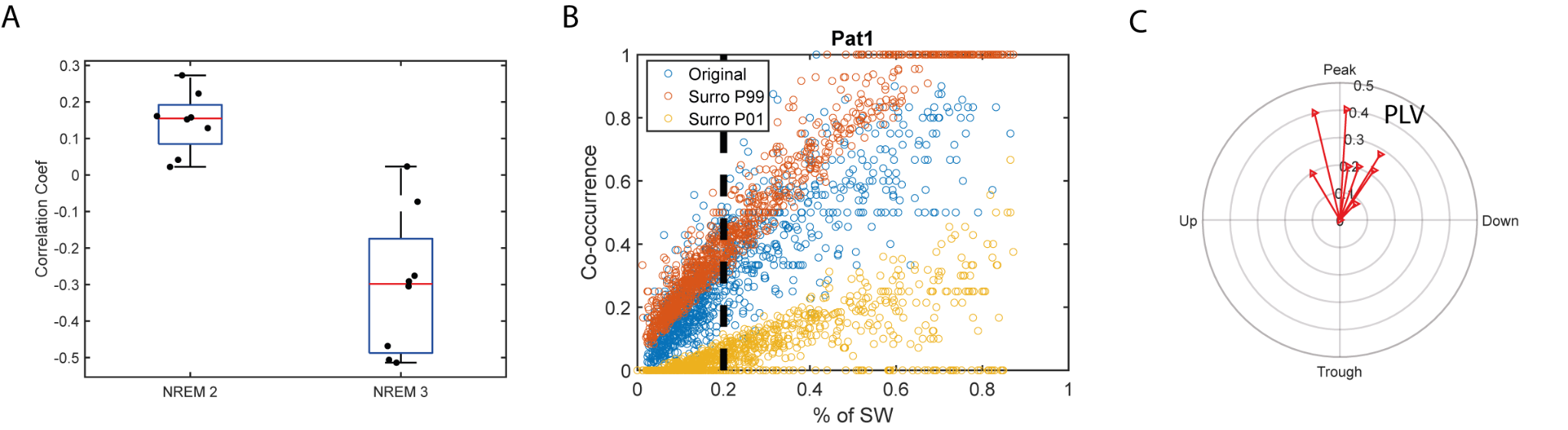


**Supplementary Figure 15. Correlation between slow wave activity (SWA, [1-4Hz]) and SIGMA power ([12-16Hz]). a**: Correlation coefficient between the SWA and SIGMA power during the non-rapid eye movement stage 2 ( N2) and the N3.**b**: Scatter plot between the co-occurrence between spindles and slow waves and the amount of slow waves over a 10-minute period. The 99 and 1 percentiles of the surrogates are also plotted. Each dot corresponds to 10 minutes of N2 or N3.**c**: Phase locking value (PLV) between the spindle center and the phase of the slow waves for all eight patients.


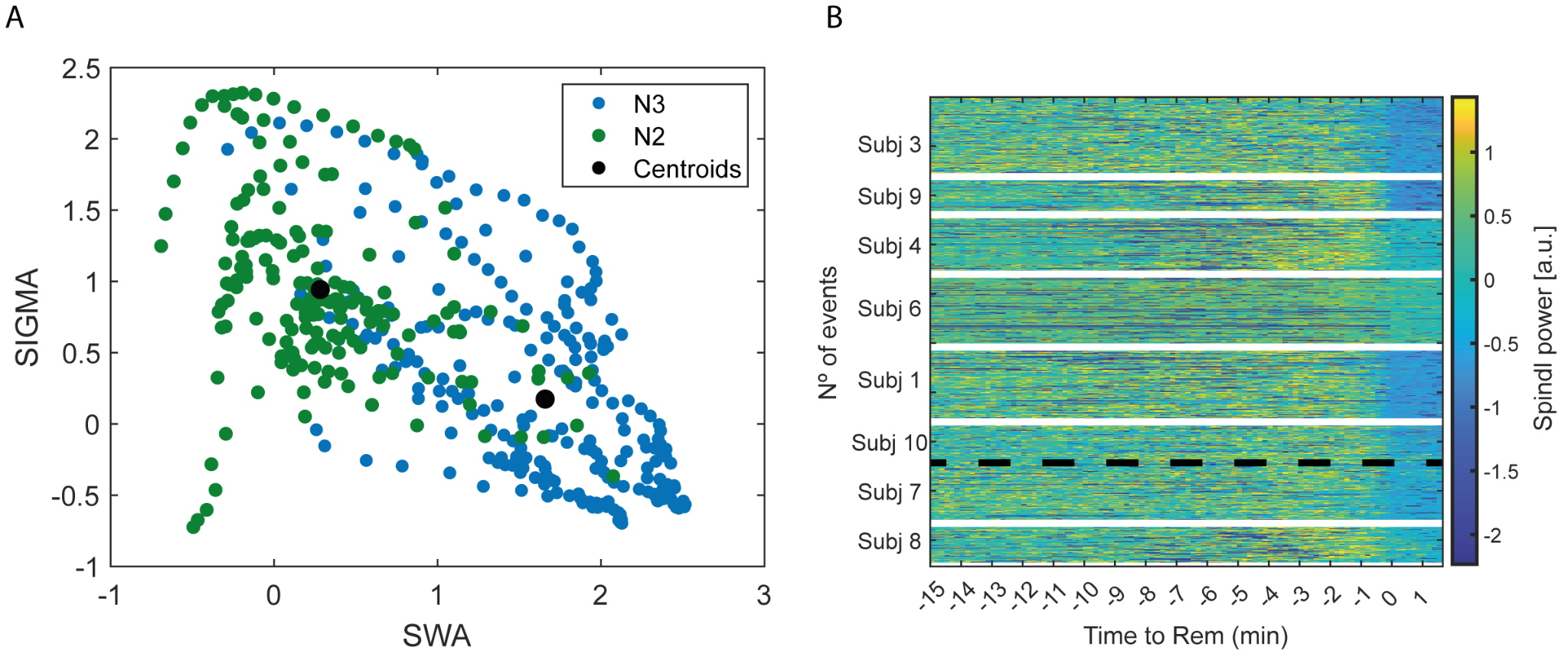
**Supplementary Figure 16. Sleep stage dynamics. a)** Cycle of the transition to rapid-eye movement sleep (REM) shown in Fig.4d with the distinction of Non-REM sleep stage 3 ( N3) and N2 and its centroids. **b)** Spindle power for all transitions to REM for all subjects.

**Spindle variation and generalized model performance**

Using our GLM model to predict the REM onset, we also computed the calibration diagram of our model and the trivial prediction. In sFig.17a, we see that our model is also better at calibrating the forecast, meaning that the probability we predict is closer to the observed one.


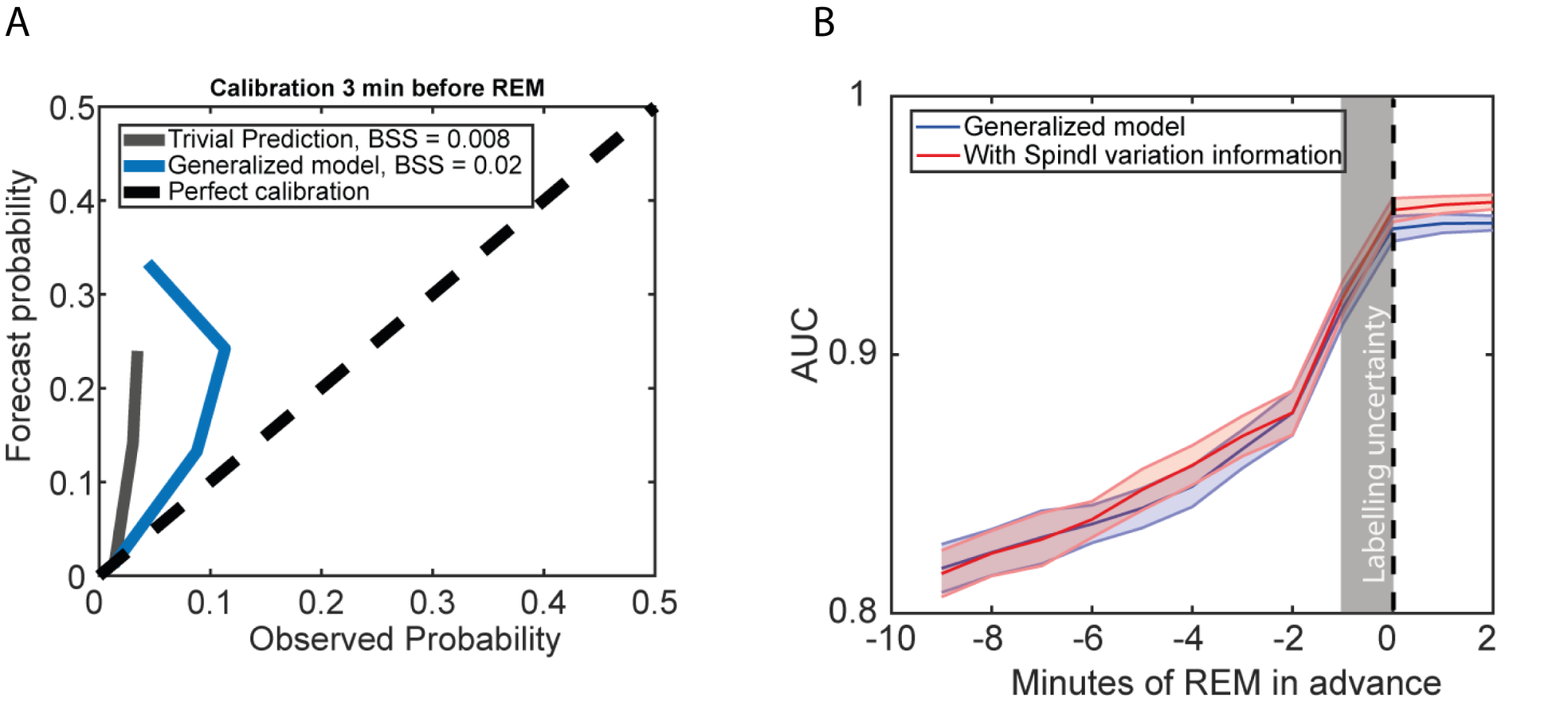


**Supplementary Figure 17. a**. Calibration diagram between the trivial and the generalized model for the rapid-eye movement (REM) prediction 3 minutes before onset. **b**. Difference of the area under the ROC curve (AUC) between using the 5 frequency bands (blue) and using the 5 frequency bands plus the information of the spindles variation.


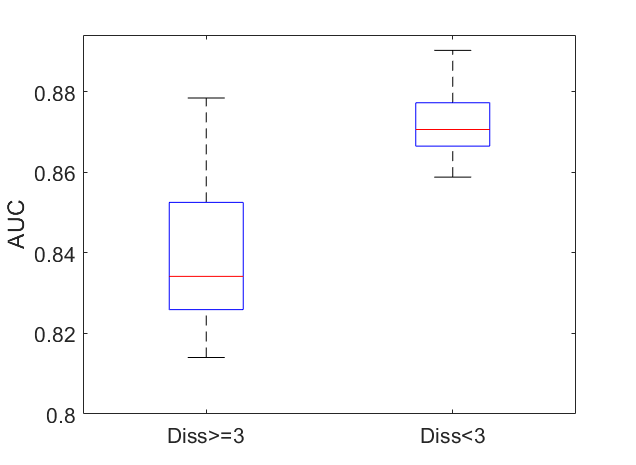


**Supplementary Figure 18. Nights closer to the canonical night are more predictable..** Boxplot of the area under the ROC curve (AUC) predicting rapid-eye movement (REM) three minutes in advance in nights with high (D>=3) and low (D<3) dissimilarity.
